# Supplementary material for: Analysing pneumococcal invasiveness using Bayesian models of pathogen progression rates
Source: PLoS Comput Biol. 2022 Feb 17;18(2):e1009389. doi: 10.1371/journal.pcbi.1009389 (PMC8901055; doi:10.1371/journal.pcbi.1009389)
Supplement: S1 Text — (DOCX) [file pcbi.1009389.s001.docx]

**Text S1: Validation of model fits using simulated data**

Simulated data were generated for 20 types of a microbe, each sampled across 20 studies. These were used to test the eight models that could be applied to typed microbial populations: the null Poisson model, the null negative binomial model, the type-specific Poisson model, the type-specific negative binomial model, the study-adjusted Poisson model, the study-adjusted negative binomial model, the study-adjusted type-specific Poisson model, and the study-adjusted type-specific negative model.

The carriage frequency of each type *j* in each study *i*, *ρ_i,j_*, was randomly generated from the distribution:

$$\rho_{i,j}\sim U(0,0.05)$$

This ensured the sum of *ρ_i,j_* within a study remained below one. The progression rates were either set to a single fixed value of *ν* across all *j* (null and study-adjusted models), else a value of *ν_j_* was selected for each type *j* (type-specific and study-adjusted type-specific models). All values of *ν* (or *ν_j_*) were randomly drawn from the distribution:

$${log}_{10}\left( \nu\right)\sim U(-5,-1)$$

This was truncated relative to the full range of values allowed by the model fit, to ensure the estimation of 95% credibility intervals were unlikely to be affected by the prior distribution boundaries.

Across studies, the number of carriage samples, *η_i_*, was drawn from a Normal distribution, rounded to the nearest integer:

$$\eta_{i}\sim\left[ N(1000,250) \right]$$

The sizes of the populations under surveillance for disease *N_i_* was set to be more variable, as observed in the actual data (Table S1):

$${log}_{10}(N_{i})\sim U(4,6)$$

For the study-adjusted models, the study scaling parameter *γ_i_* was set to one for *i*=1. For the other studies, *γ_i_* was randomly drawn from the distribution:

$${log}_{10}(\gamma_{i})\sim N(0,2)$$

For all models using a negative binomial distribution, the precision parameter ϕ was set to 0.1.

For all eight tested models, counts of carriage isolates of each type in each study (*c_i,j_*) were generated from the distribution:

$$c_{i,j}\sim Binom(\rho_{i,j},\eta_{i})$$

For each tested model, the counts of disease isolates of each type in each study (*d_i,j_*) were generated from the Poisson or negative binomial distribution, using the appropriate calculation for 𝔼[*d_i,j_*].

All eight models were fitted to data simulated from each model, using two MCMCs of 10^4^ iterations. Bayes factors were used to compare the model fits to each dataset using bridge sampling run for 10^4^ iterations.
