# Supplementary material for: Analysing pneumococcal invasiveness using Bayesian models of pathogen progression rates
Source: PLoS Comput Biol. 2022 Feb 17;18(2):e1009389. doi: 10.1371/journal.pcbi.1009389 (PMC8901055; doi:10.1371/journal.pcbi.1009389)
Supplement: S2 Text — (DOCX) [file pcbi.1009389.s002.docx]

**Text S2: Sample descriptions for genomic and genotyped datasets**

The *S. pneumoniae* population was previously divided into strains by the Global Pneumococcal Sequencing project [1] using PopPUNK [2]. Previous estimation of the effect of strain background on invasiveness by Gladstone *et al* [3] used two study populations. The first combined two carriage studies from Agincourt and Soweto, in South Africa, with nationwide surveillance of IPD by the National Institute for Communicable Diseases. After excluding carriage and disease isolates from individuals reported as being HIV positive, genomes were available for 1,089 carriage isolates and 855 IPD isolates from children under seven years old. Model fitting required calculating the number of swabs taken from HIV negative children under seven in the carriage studies. In Soweto, the reported age stratification meant this number could only be calculated for children under five [4], and therefore four carriage isolates from children aged five or six were excluded from the study. In Agincourt, the reported age stratification meant 33 isolates from children aged six had to be excluded [5]. As the prevalence of HIV was low in infants in the Agincourt study [6], and the exact number of swabs from HIV positive children was not reported, no adjustment was made to the sampling based on HIV status. The population of children under surveillance for IPD was estimated from nationwide census data between 2009-2013 [7]. All samples were stratified into the post-PCV7 (2009-2011) and post-PCV13 (2012-2013) periods [1].

The second study population combined a carriage survey from Massachusetts, USA and isolates from the Active Bacterial Core Surveillance (ABCS) of IPD across several states. Isolates were collected over the sampling periods of 2000-2001, 2003-2004, 2006-2007 and 2008-2009. As only 51 IPD samples were available for the earliest two periods, and the population was changing rapidly over these years [8], only samples from the more stable post-PCV7 2006-2009 period were studied [9]. This included 405 IPD isolates, modelled as coming from a time interval of two years (as samples only came from 2007 and 2009) from a population size calculated from the ABCS population matrix [10]. Of the carriage isolates, 280 were assigned to strains from genomic data, while 291 were only associated with multi-locus sequence typing (MLST) genotypes [11]. Gladstone *et al* assigned these isolates to strains using the close relationship between MLST and PopPUNK population classifications [2,3].

As these studies were estimating odds ratios across samples stratified by year, Gladstone *et al* randomly subsampled isolates to ensure an even ratio of disease and carriage at each sampling timepoint [3]. This correction is unnecessary with the models described in this study, as the γ_i_ scaling parameters can adjust for differing levels of surveillance between samples (incorporating variation in both the samples collected, and the subset sequenced). Furthermore, such subsampling would distort the inferred *ρ_i,x_*.

MLST genotypes were also available for isolates from the studies of serotype invasiveness from Stockholm [12], Finland [13,14] and Oxford [15]. Where possible, these were assigned to GPS strains, either using the isolate’s MLST sequence type, or the clonal complex to which this type belonged at the time of publication [16]. If an isolate could not be assigned to a GPS strain, then its MLST sequence type was used to designate its genotype instead. Isolates that could not be unequivocally assigned a strain, serotype and source (carriage or disease) were omitted.

For the Oxford study, these criteria meant 84 of the 150 disease isolates, and 113 of the 351 carriage isolates, could be analysed. The carriage isolates were assembled from three different surveys, only one of which could be linked to a more detailed published description of the total carriage sample size [17]. As the isolates in this study were recovered from 213 individuals, and the Oxford study attempted to minimise multiple sampling from any one host [15], the overall carriage sample size for the three studies was approximated as three times the number of individuals in this published study (639).

For the Finland study, the criteria described above meant 143 of the 224 disease isolates, and 102 of the 217 carriage isolates, could be analysed. The carriage isolates were recovered from ten longitudinal samples from each of 329 individuals [18]. As the Finland study sought to avoid multiple sampling of the same carriage episode [14], the host population size was used as the carriage sample size.

For the Stockholm study, the criteria described above meant 65 of the 165 disease isolates, and 178 of the 550 carriage isolates, could be analysed. The carriage isolates were recovered from studies conducted between 1997 and 2004. Two published descriptions of the original carriage studies reported the recovery of 506 isolates from 1,330 individuals [19,20]. As this accounted for over 90% of the nasopharyngeal isolates, this number was used as the carriage sample size.

An additional study from Portugal used a collection of disease isolates primarily recovered from adults [21]. Applying the same criteria as to the child disease studies allowed unambiguous assignation of genotype and serotype to 463 of the 769 carriage isolates, and 152 of the 475 of the disease isolates. The carriage isolates were a subset of a previous study [22], and originated from 1,170 nasopharyngeal swabs (R. Sá-Leão, personal communication).

**References**

1. Lo SW, Gladstone RA, van Tonder AJ, Lees JA, du Plessis M, Benisty R, et al. Pneumococcal lineages associated with serotype replacement and antibiotic resistance in childhood invasive pneumococcal disease in the post-PCV13 era: an international whole-genome sequencing study. Lancet Infect Dis. 2019;19: 759–769. doi:10.1016/S1473-3099(19)30297-X

2. Lees JA, Harris SR, Tonkin-Hill G, Gladstone RA, Lo S, Weiser JN, et al. Fast and flexible bacterial genomic epidemiology with PopPUNK. Genome Res. 2019;29: 304–316. doi:10.1101/360917

3. Gladstone RA, Lo SW, Lees JA, Croucher NJ, van Tonder AJ, Corander J, et al. International genomic definition of pneumococcal lineages, to contextualise disease, antibiotic resistance and vaccine impact. EBioMedicine. 2019;43: 338–346. doi:10.1016/j.ebiom.2019.04.021

4. Nzenze SA, Von Gottberg A, Shiri T, Van Niekerk N, De Gouveia L, Violari A, et al. Temporal Changes in Pneumococcal Colonization in HIV-infected and HIV-uninfected Mother-Child Pairs Following Transitioning from 7-valent to 13-valent Pneumococcal Conjugate Vaccine, Soweto, South Africa. J Infect Dis. 2015. doi:10.1093/infdis/jiv167

5. Madhi SA, Nzenze SA, Nunes MC, Chinyanganya L, Van Niekerk N, Kahn K, et al. Residual colonization by vaccine serotypes in rural South Africa four years following initiation of pneumococcal conjugate vaccine immunization. Expert Rev Vaccines. 2020;19: 383–393. doi:10.1080/14760584.2020.1750377

6. Nzenze SA, Shiri T, Nunes MC, Klugman KP, Kahn K, Twine R, et al. Temporal changes in pneumococcal colonization in a rural African community with high HIV prevalence following routine infant pneumococcal immunization. Pediatr Infect Dis J. 2013;32: 1270–1278. doi:10.1097/01.inf.0000435805.25366.64

7. Statistics South Africa. Mid-year population estimates: 2009-2013. 2013.

8. Croucher NJ, Finkelstein JA, Pelton SI, Mitchell PK, Lee GM, Parkhill J, et al. Population genomics of post-vaccine changes in pneumococcal epidemiology. Nat Genet. 2013;45: 656–663. doi:10.1038/ng.2625

9. Chang Q, Stevenson AE, Croucher NJ, Lee GM, Pelton SI, Lipsitch M, et al. Stability of the pneumococcal population structure in Massachusetts as PCV13 was introduced. BMC Infect Dis. 2015;15: 68. doi:10.1186/s12879-015-0797-z

10. Centres for Disease Prevention and Control. Active Bacterial Core surveillance matrix. 2018. Available: https://www.cdc.gov/abcs/downloads/abcs-surveillance-matrix.pdf

11. Enright MC, Spratt BG. A multilocus sequence typing scheme for *Streptococcus pneumoniae*: Identification of clones associated with serious invasive disease. Microbiology. 1998;144: 3049–3060. doi:10.1099/00221287-144-11-3049

12. Browall S, Backhaus E, Naucler P, Galanis I, Sjöström K, Karlsson D, et al. Clinical manifestations of invasive pneumococcal disease by vaccine and non-vaccine types. Eur Respir J. 2014;44: 1646–57. doi:10.1183/09031936.00080814

13. Syrjänen RK, Kilpi TM, Kaijalainen TH, Herva EE, Takala AK. Nasopharyngeal carriage of *Streptococcus pneumoniae* in Finnish children younger than 2 years old. J Infect Dis. 2001;184: 451–459. doi:10.1086/322048

14. Hanage WP, Kaijalainen TH, Syrjänen RK, Auranen K, Leinonen M, Mäkelä PH, et al. Invasiveness of serotypes and clones of *Streptococcus pneumoniae* among children in Finland. Infect Immun. 2005;73: 431–5. doi:10.1128/IAI.73.1.431-435.2005

15. Brueggemann AB, Griffiths DT, Peto T, Meats E, Crook DW, Spratt BG. Clonal Relationships between Invasive and Carriage *Streptococcus pneumoniae* and Serotype‐ and Clone‐Specific Differences in Invasive Disease Potential. J Infect Dis. 2003;187: 1424–32. doi:10.1086/374624

16. Feil EJ, Enright MC. Analyses of clonality and the evolution of bacterial pathogens. Curr Opin Microbiol. 2004;7: 308–313. doi:10.1016/j.mib.2004.04.002

17. Sleeman KL, Daniels L, Gardiner M, Griffiths D, Deeks JJ, Dagan R, et al. Acquisition of *Streptococcus pneumoniae* and nonspecific morbidity in infants and their families: A cohort study. Pediatr Infect Dis J. 2005;24: 121–127. doi:10.1097/01.inf.0000151030.10159.b1

18. Hanage WP, Auranen K, Syrjanen R, Herva E, Makela PH, Kilpi T, et al. Ability of pneumococcal serotypes and clones to cause acute otitis media: implications for the prevention of otitis media by conjugate vaccines. Infect Immun. 2004;72: 76–81.

19. Normark BH, Christensson B, Sandgren A, Noreen B, Sylvan S, Burman LG, et al. Clonal Analysis of *Streptococcus pneumoniae* Nonsusceptible to Penicillin at Day-Care Centers with Index Cases, in a Region with Low Incidence of Resistance: Emergence of an Invasive Type 35B Clone among Carriers. Microb Drug Resist. 2003;9: 337–344. doi:10.1089/107662903322762761

20. Galanis I, Lindstrand A, Darenberg J, Browall S, Nannapaneni P, Sjöström K, et al. Effects of PCV7 and PCV13 on invasive pneumococcal disease and carriage in Stockholm, Sweden. Eur Respir J. 2016;47: 1208–1218. doi:10.1183/13993003.01451-2015

21. Sá-Leao R, Pinto F, Aguiar S, Nunes S, Carriço JAJA, Frazao N, et al. Analysis of invasiveness of pneumococcal serotypes and clones circulating in Portugal before widespread use of conjugate vaccines reveals heterogeneous behavior of clones expressing the same serotype. J Clin Microbiol. 2011;49: 1369–75. doi:10.1128/jcm.01763-10

22. Mato R, Santos Sanches I, Simas C, Nunes S, Carriço JA, Sousa NG, et al. Natural history of drug-resistant clones of *Streptococcus pneumoniae* colonizing healthy children in Portugal. Microb Drug Resist. 2005;11: 309–322. doi:10.1089/mdr.2005.11.309
