# Supplementary figures and images for: Analysing pneumococcal invasiveness using Bayesian models of pathogen progression rates
Source: PLoS Comput Biol. 2022 Feb 17;18(2):e1009389. doi: 10.1371/journal.pcbi.1009389 (PMC8901055; doi:10.1371/journal.pcbi.1009389)

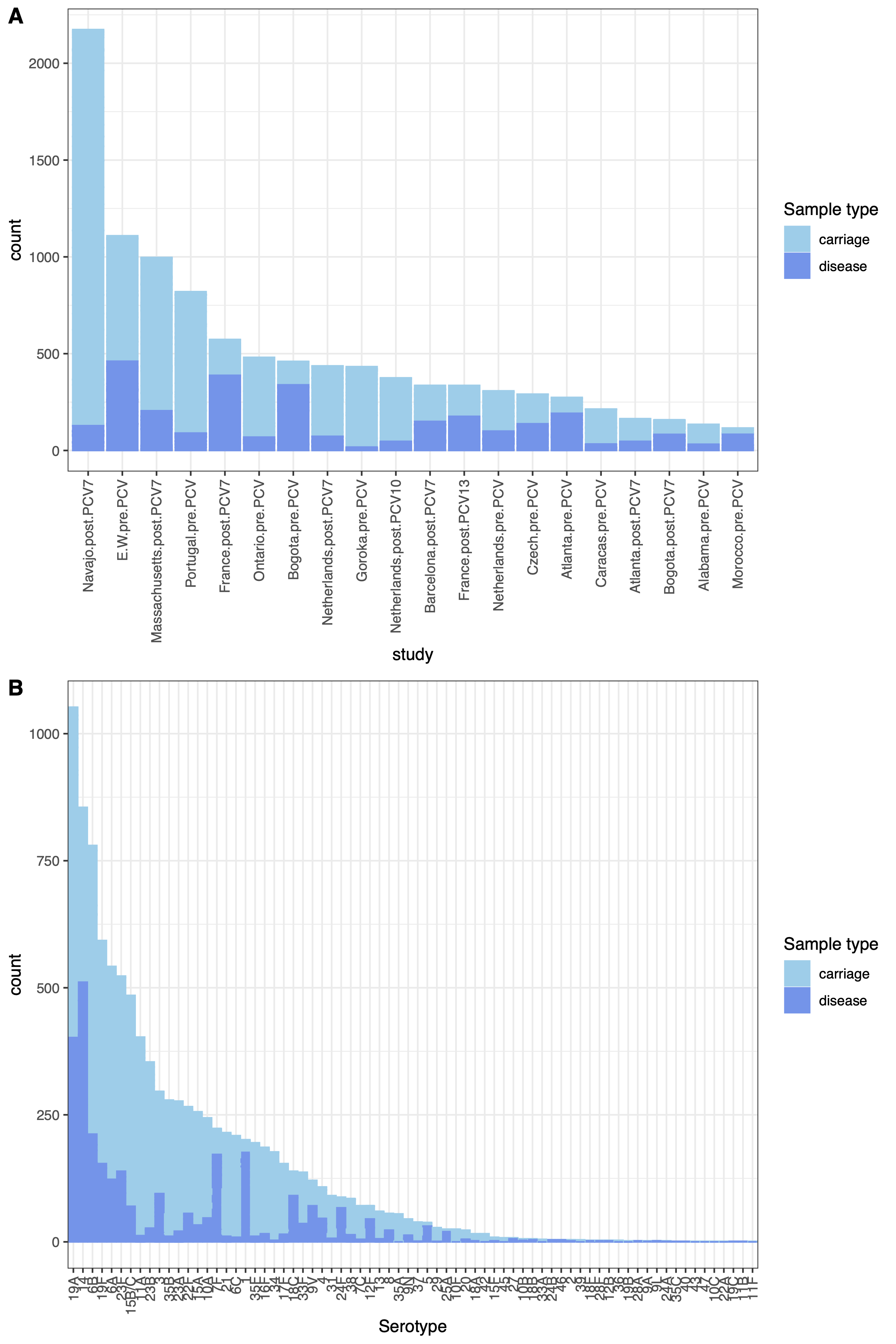

Supplement: S1 Fig — (A) Stacked bar plot showing the distribution of carriage and disease isolates between studies. (B) Stacked bar plot showing the distribution of carriage and disease isolates between serotypes. (PNG) [file pcbi.1009389.s003.png]

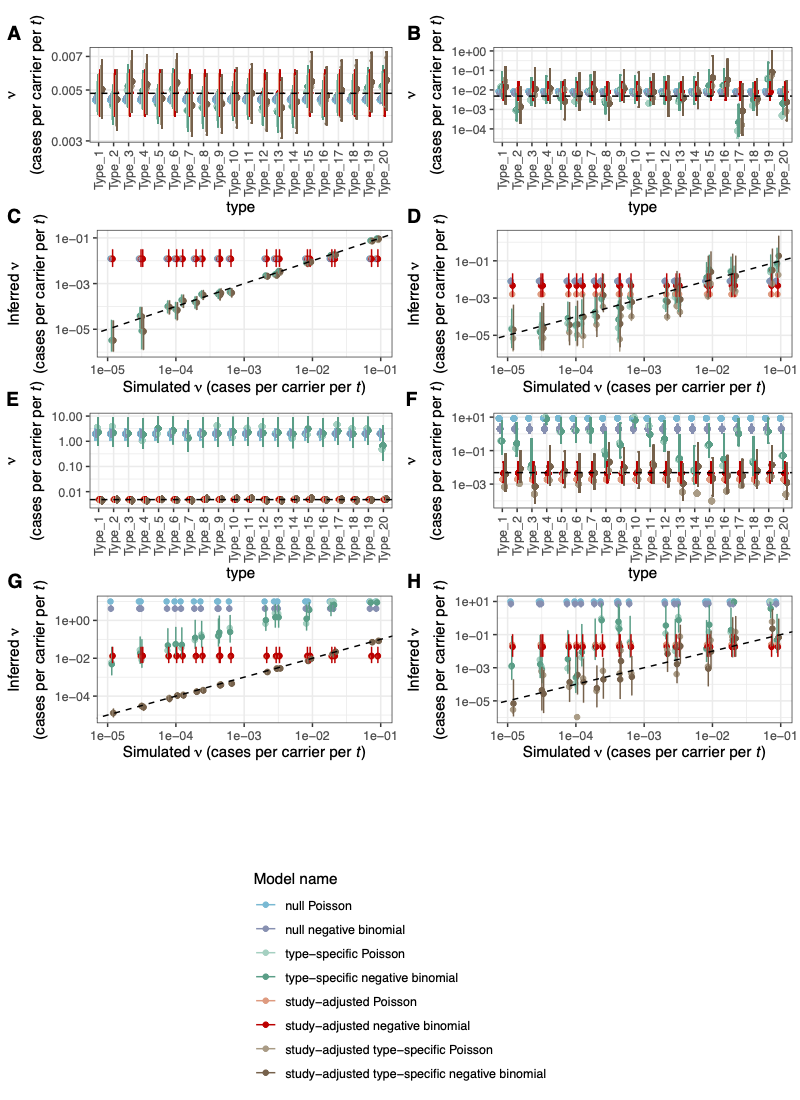

Supplement: S2 Fig — Each panel shows the fit of eight different models, indicated by colour, to data simulated from a single model structure. Points represent the median estimate, and the error bars show the 95% credible intervals. The models from which data were simulated were (A) null Poisson, (B) null negative binomial, (C) type-specific Poisson, (D) type-specific negative binomial, (E) study-adjusted Poisson, (F) study-adjusted negative binomial, (G) study-adjusted type-specific Poisson, and (H) study-adjusted type-specific negative binomial. For panels (A), (B), (E) and (F), all types had the same progression rate, and therefore the dashed horizontal line represents the single true value used in the simulations. For panels (B), (C), (G) and (H), each of the 20 types had a different progression rate, which determines the horizontal position of the point on the graph. In these four plots, the dashed line indicates the line of identity. (PNG) [file pcbi.1009389.s004.png]

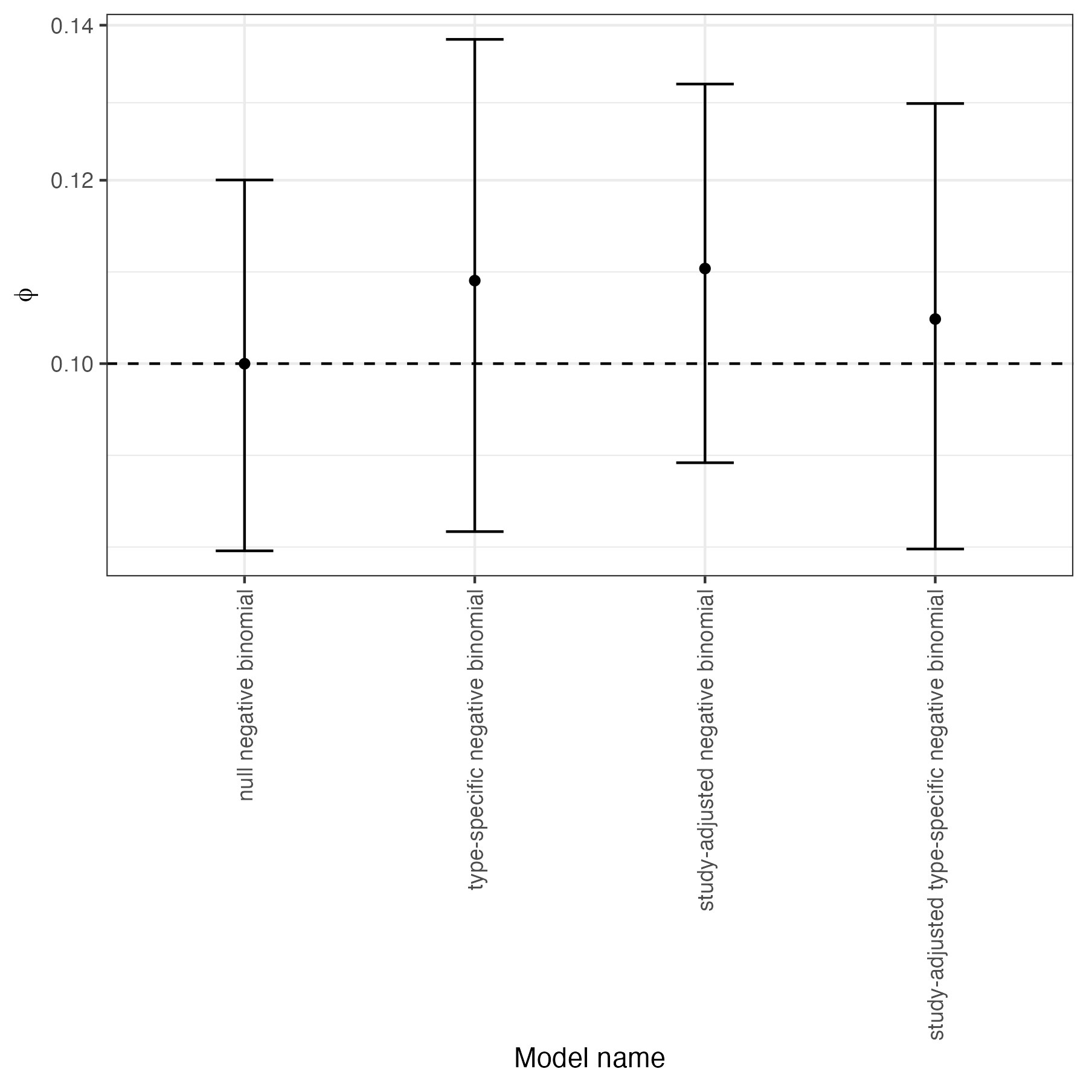

Supplement: S3 Fig — The horizontal position of the point corresponds to the model that was both used to generate the data, and to then infer the parameter value from these data. The vertical position of the point indicates the median estimate of the parameter, and the error bars represent the 95% credibility interval. The horizontal dashed line indicates the true value of the parameter used to simulate data, ϕ = 0.1. (PNG) [file pcbi.1009389.s005.png]

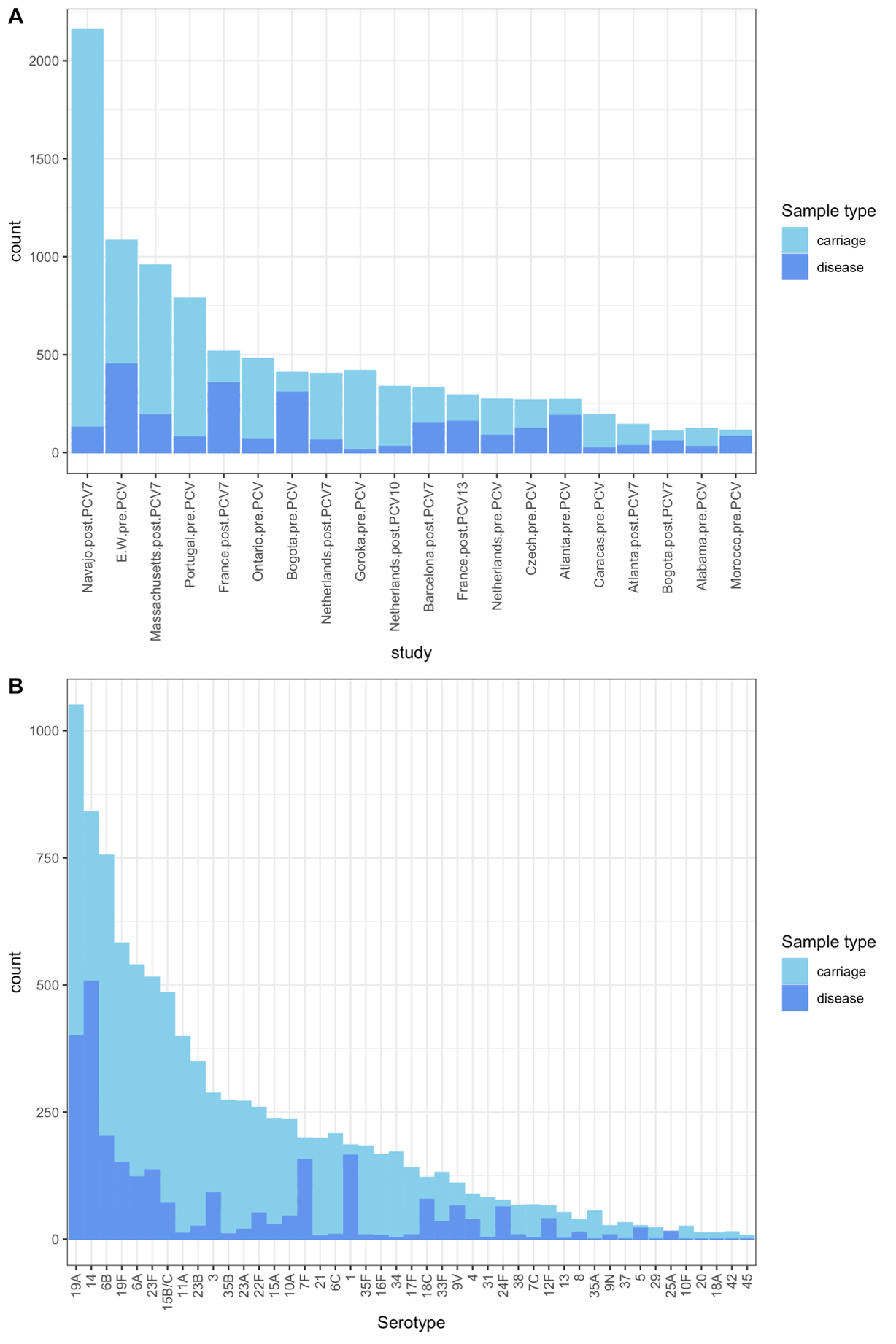

Supplement: S4 Fig — (A) Stacked bar plot showing the distribution of carriage and disease isolates between studies. (B) Stacked bar plot showing the distribution of carriage and disease isolates between serotypes. (PNG) [file pcbi.1009389.s006.png]

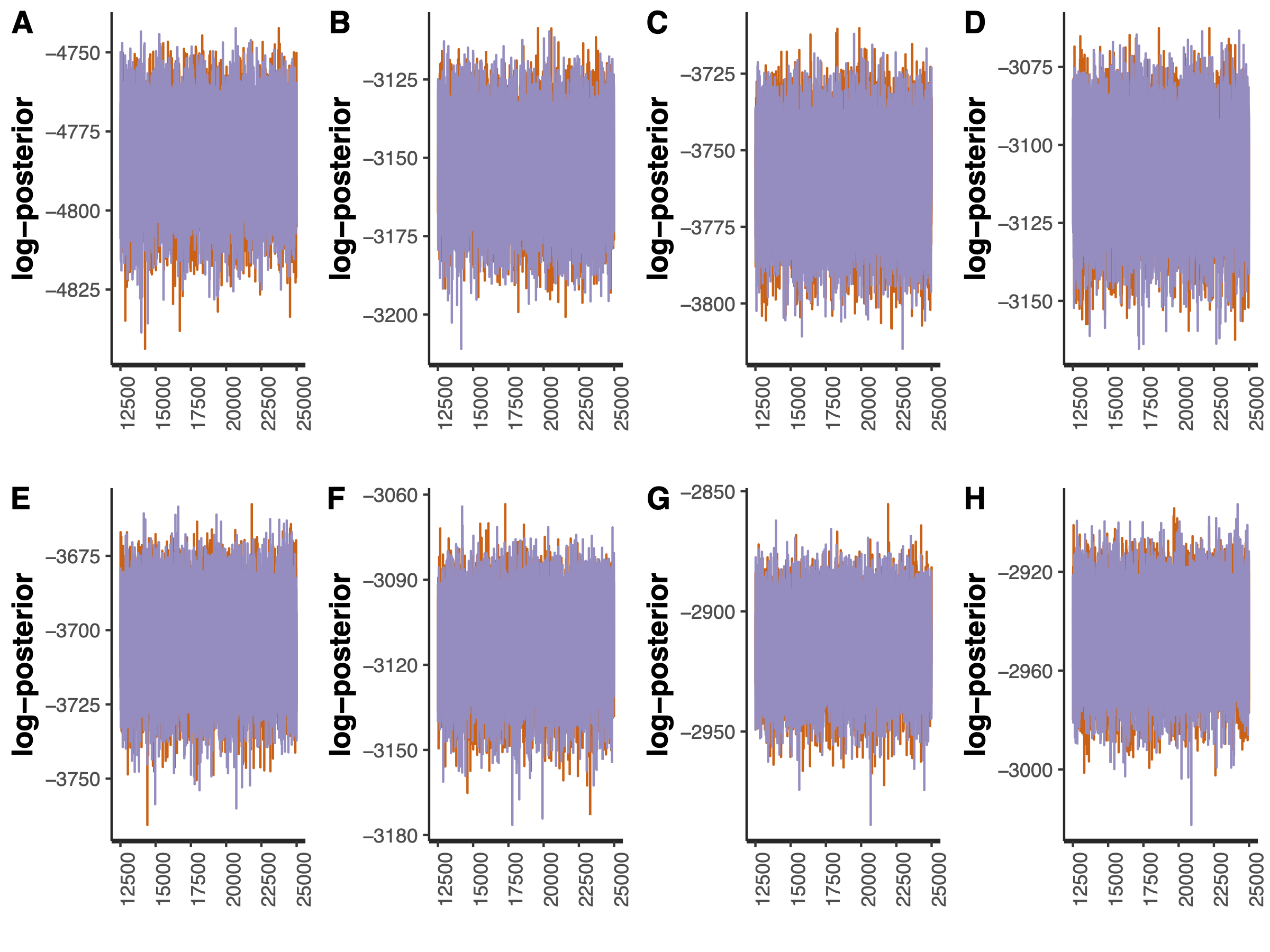

Supplement: S5 Fig — The horizontal axis shows the generation of the MCMC, with values for the two chains shown by orange and purple lines. Each panel corresponds to a different model: (A) null Poisson model; (B) null negative binomial model; (C) type-specific Poisson model; (D) type-specific negative binomial model; (E) study-adjusted Poisson model; (F) study-adjusted negative binomial model; (G) study-adjusted type-specific Poisson model; (H) study-adjusted type-specific negative binomial model. (PNG) [file pcbi.1009389.s007.png]

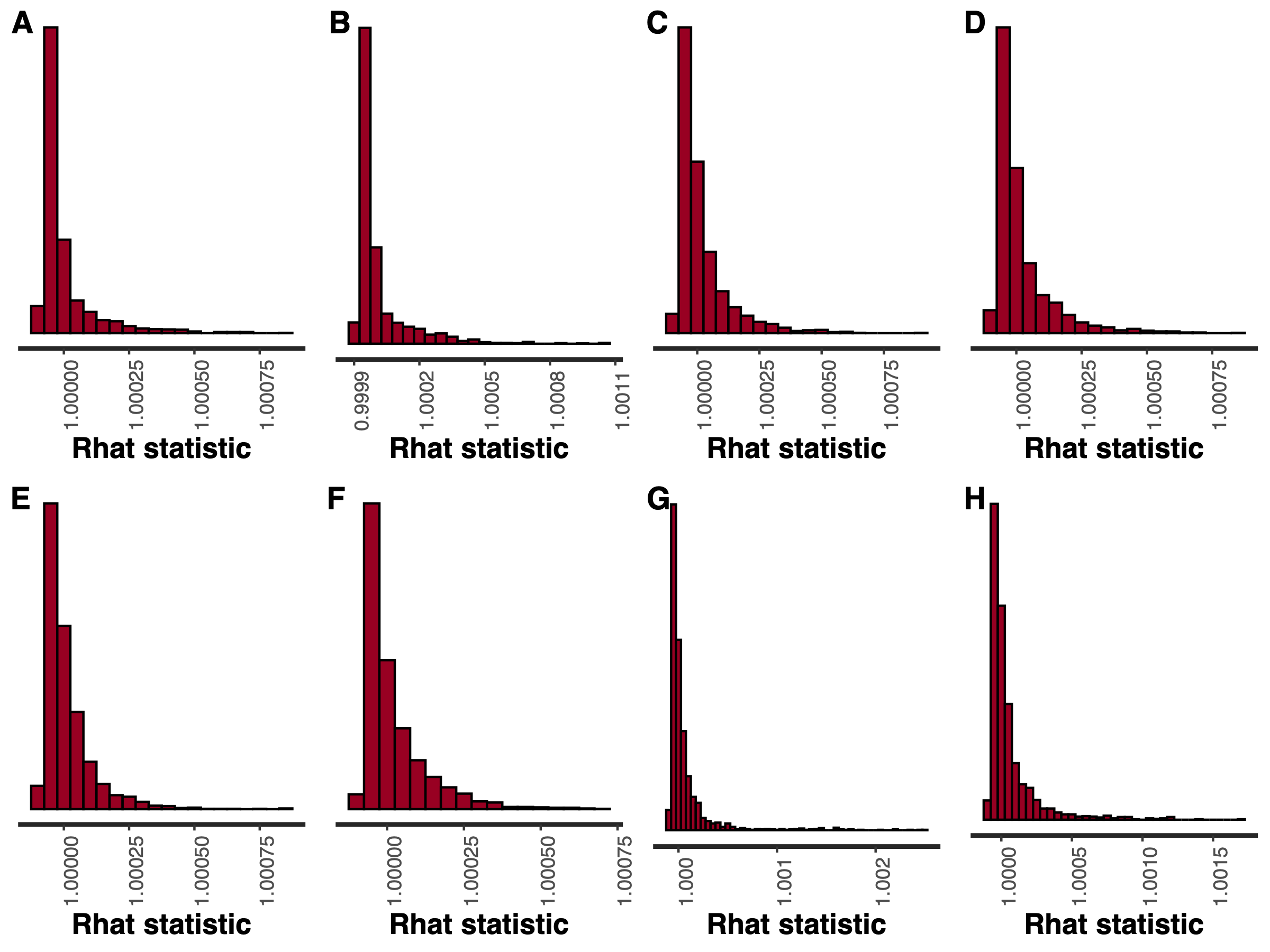

Supplement: S6 Fig — Each panel corresponds to a different model: (A) null Poisson model; (B) null negative binomial model; (C) type-specific Poisson model; (D) type-specific negative binomial model; (E) study-adjusted Poisson model; (F) study-adjusted negative binomial model; (G) study-adjusted type-specific Poisson model; (H) study-adjusted type-specific negative binomial model. (PNG) [file pcbi.1009389.s008.png]

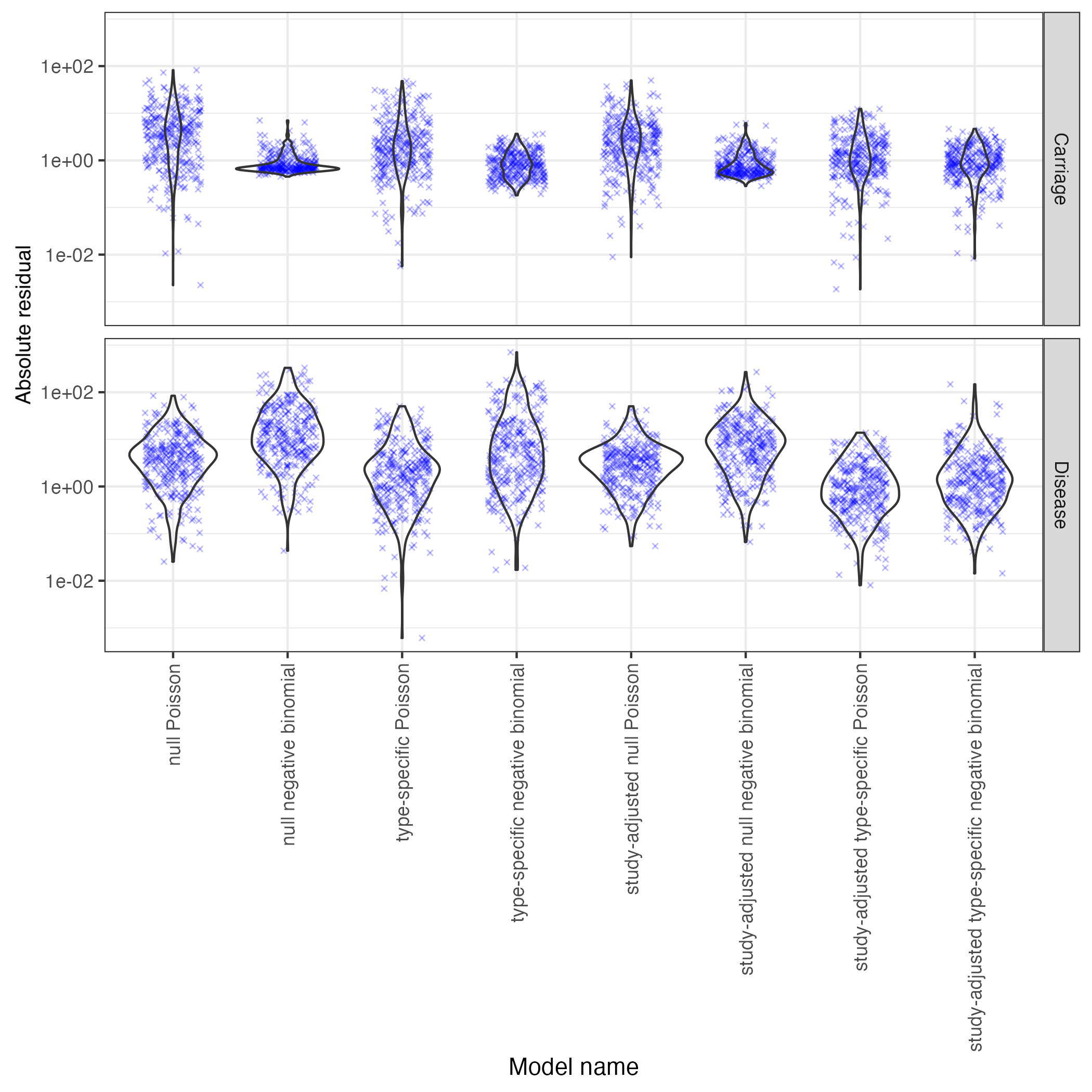

Supplement: S7 Fig — Blue crosses represent the individual observations. (PNG) [file pcbi.1009389.s009.png]

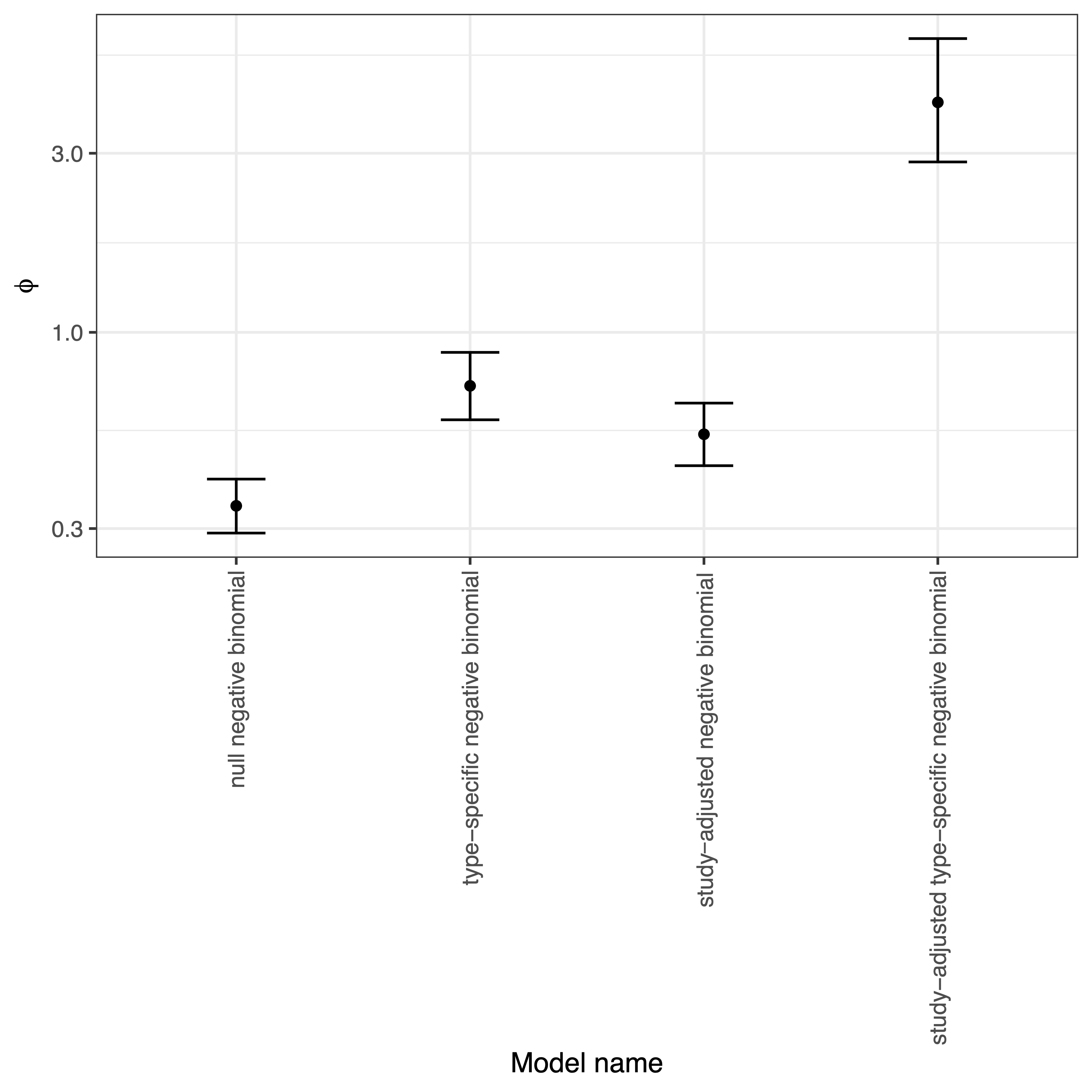

Supplement: S8 Fig — The points represent the median estimates from the MCMCs, and the error bars show the 95% credibility intervals. (PNG) [file pcbi.1009389.s010.png]

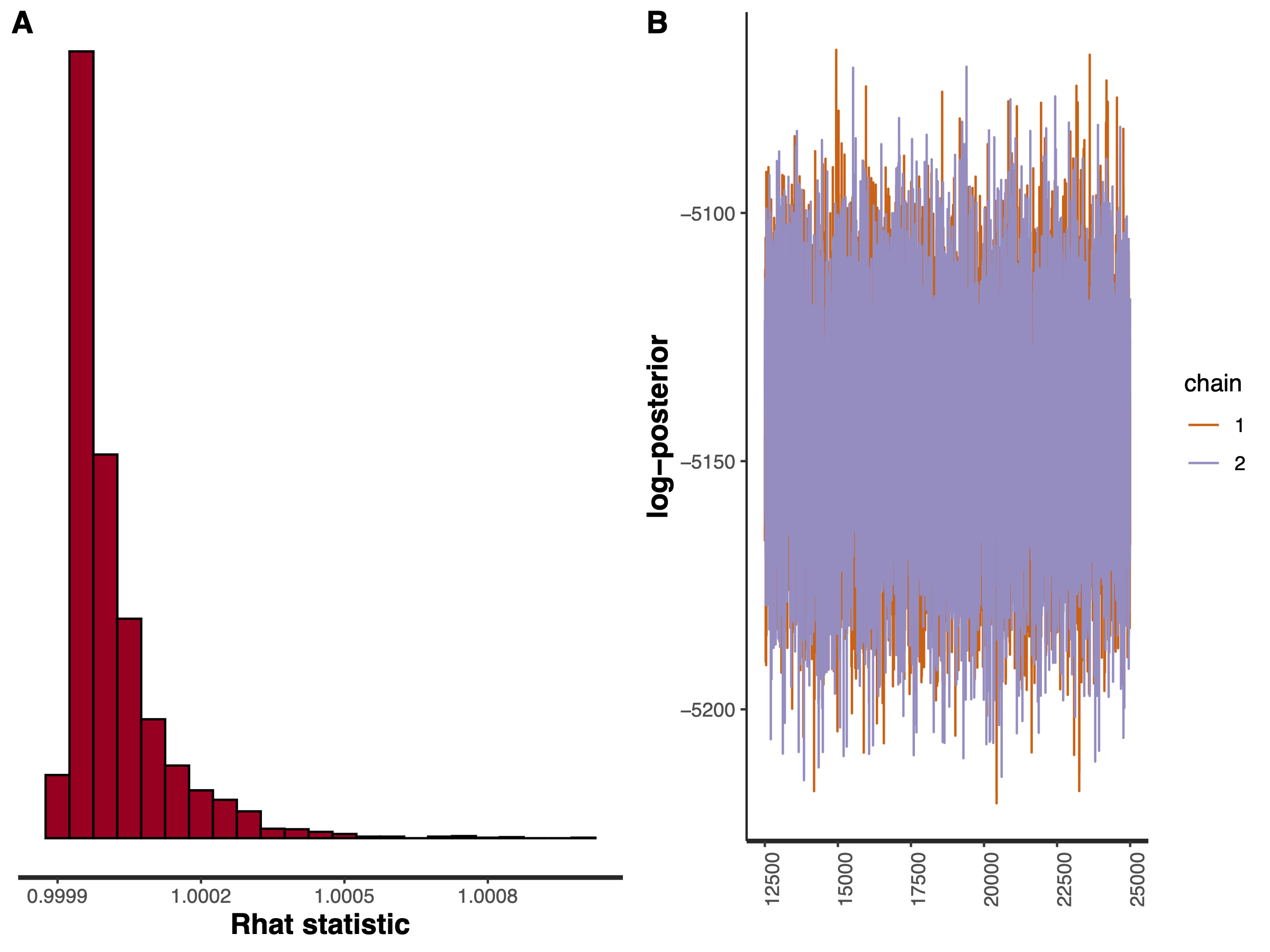

Supplement: S9 Fig — (A) Histogram showing the distribution of R^ values. (B) Post-warmup MCMC traces of the log posterior probability. (PNG) [file pcbi.1009389.s011.png]

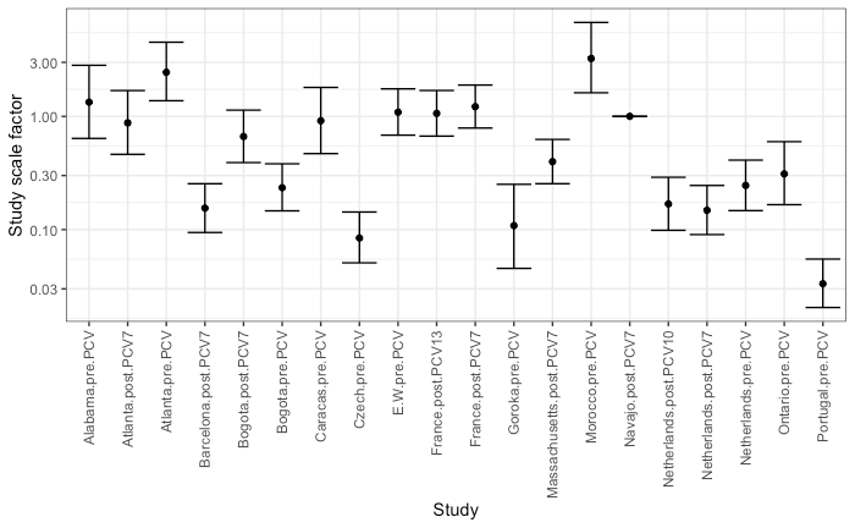

Supplement: S10 Fig — The reference study, for which the value was fixed at one, was the Navajo post-PCV7 dataset, which had the greatest sample size in this meta-analysis (S1 Fig). (PNG) [file pcbi.1009389.s012.png]

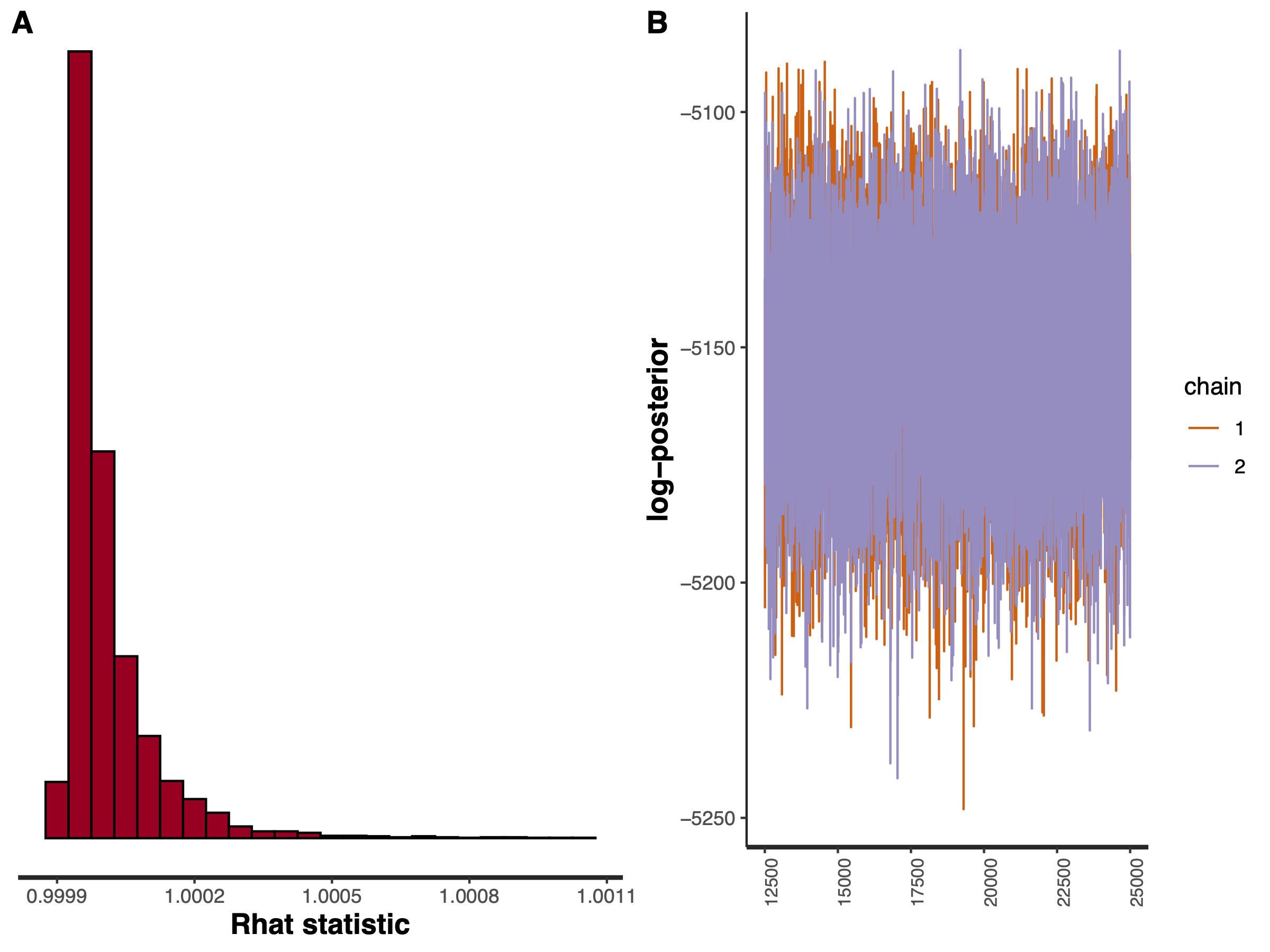

Supplement: S11 Fig — (A) Histogram showing the distribution of R^ values. (B) Post-warmup MCMC traces of the log posterior probability. (PNG) [file pcbi.1009389.s013.png]

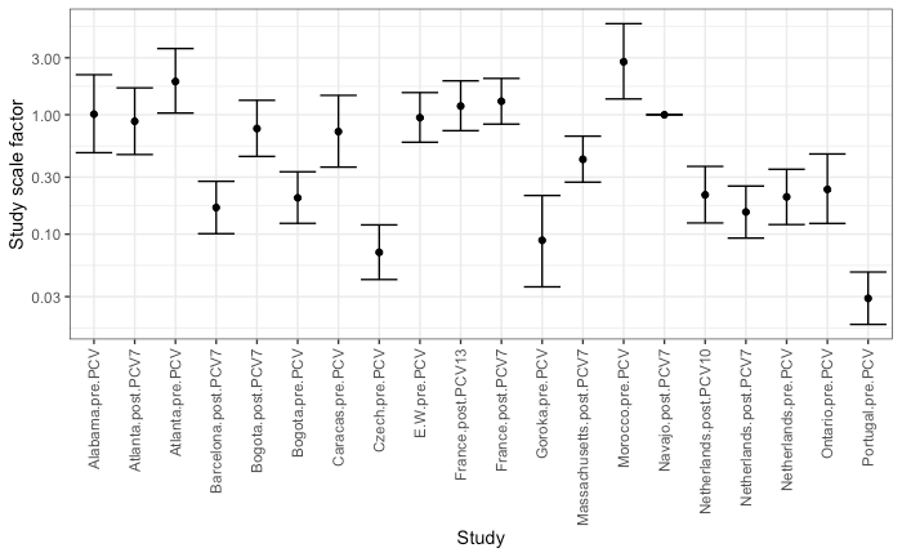

Supplement: S12 Fig — The reference study, for which the value was fixed at one, was the Navajo post-PCV7 dataset, which had the greatest sample size in this meta-analysis (S1 Fig). (PNG) [file pcbi.1009389.s014.png]

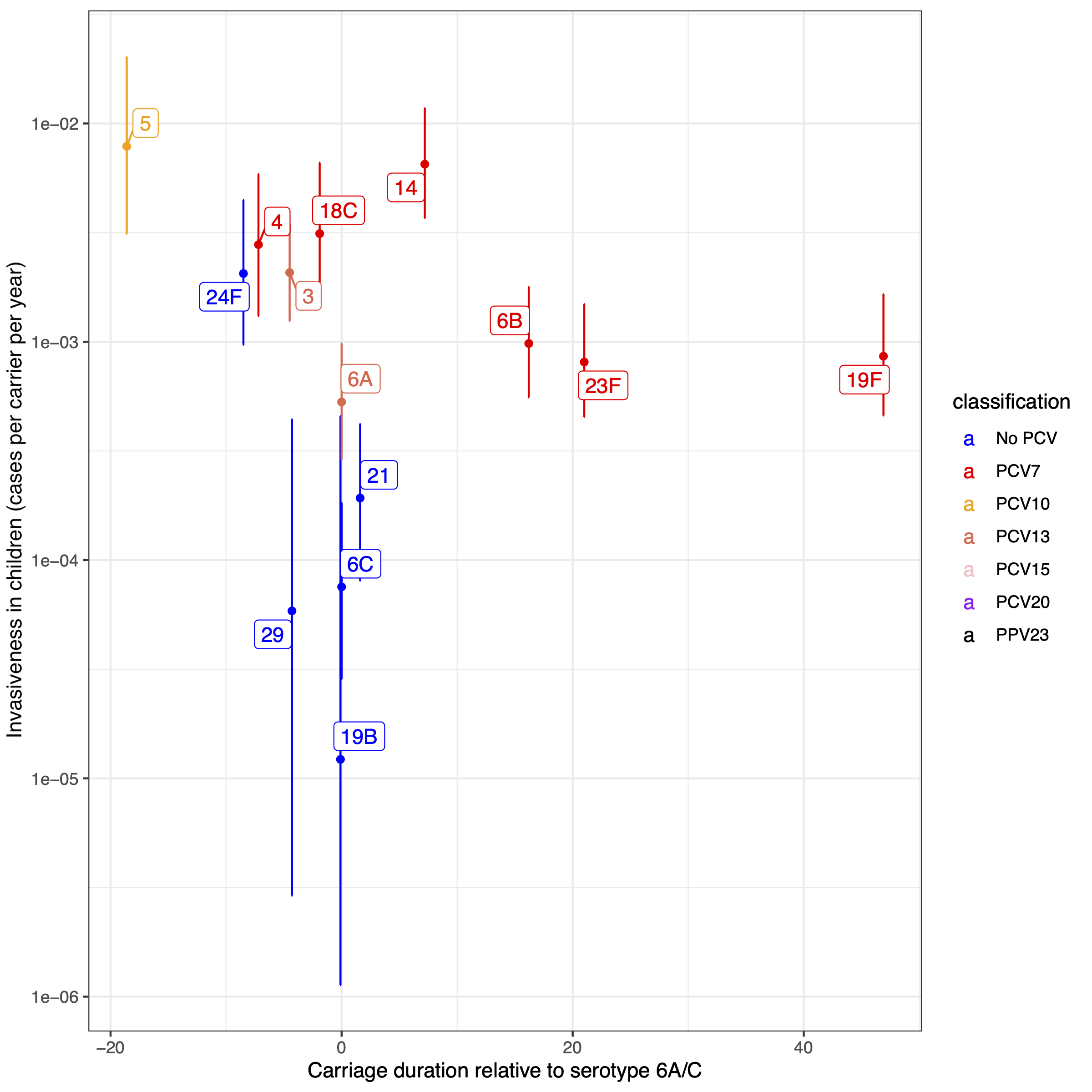

Supplement: S13 Fig — The carriage duration estimates were derived from a multi-variate lasso regression that included both serotype and antibiotic resistance phenotypes. Values were available for 14 serotypes, all relative to the carriage duration of serotype 6A/C, which was assigned a value of zero days. (PNG) [file pcbi.1009389.s015.png]

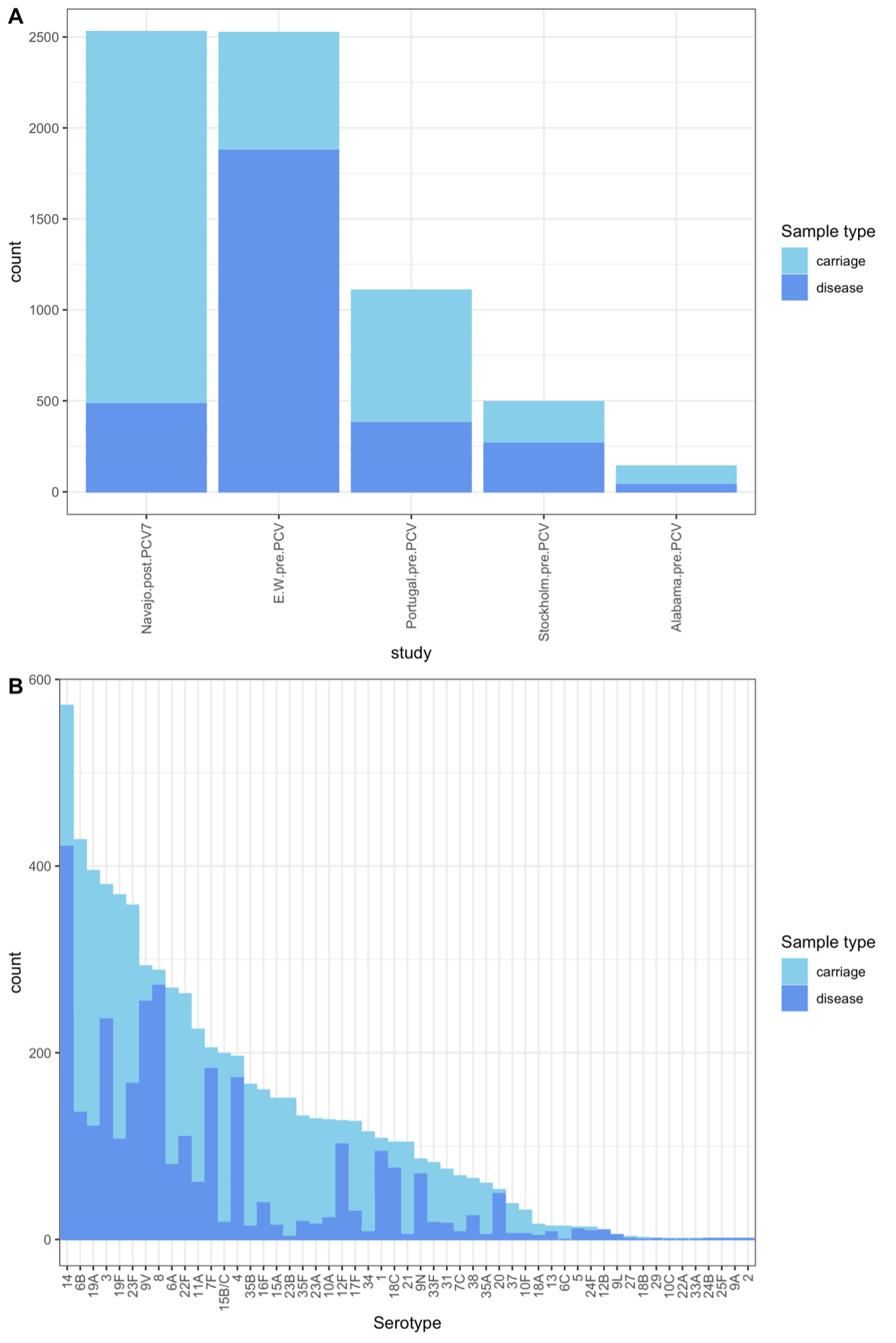

Supplement: S14 Fig — (A) Stacked bar plot showing the distribution of carriage and disease isolates between studies. (B) Stacked bar plot showing the distribution of carriage and disease isolates between serotypes. (PNG) [file pcbi.1009389.s016.png]

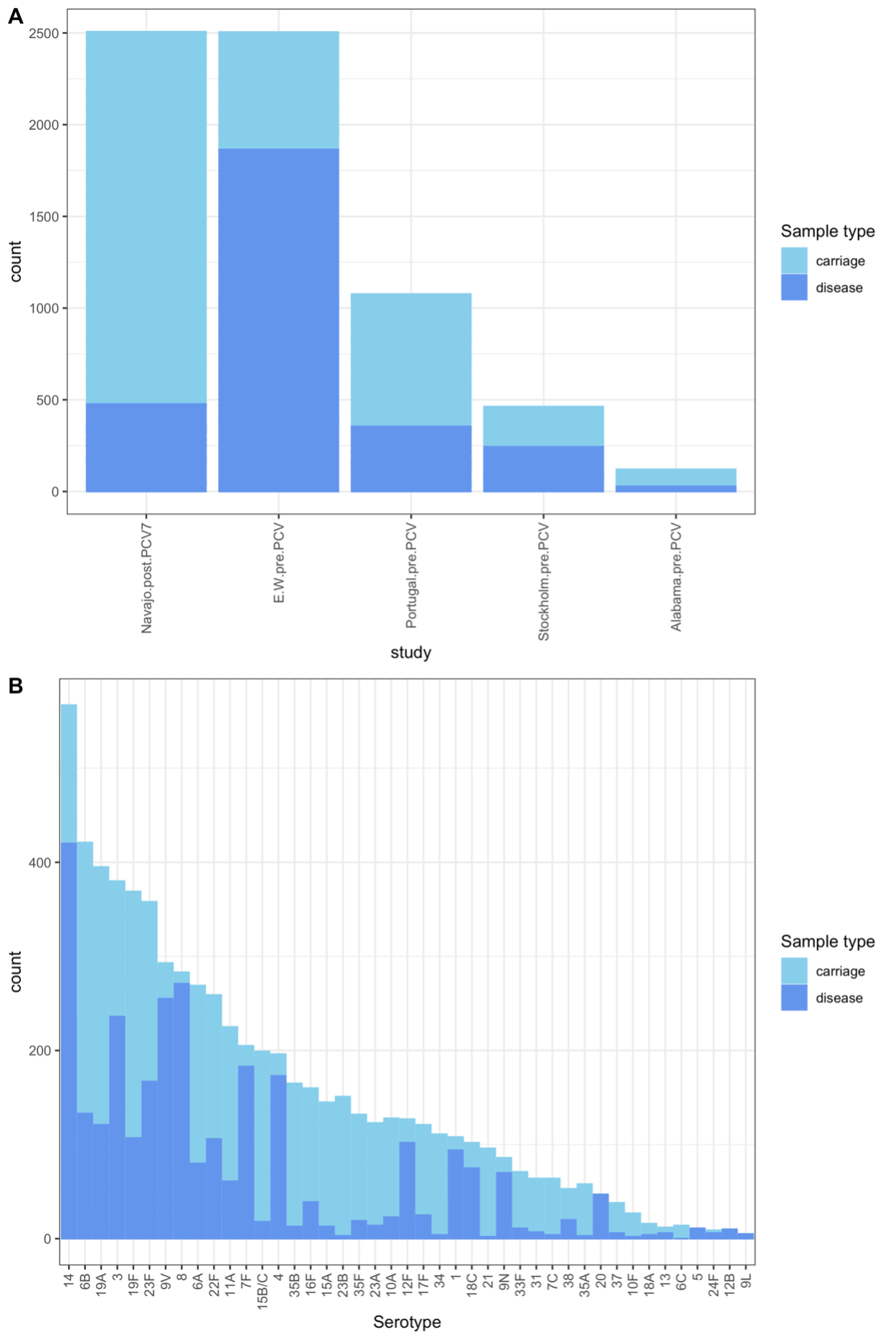

Supplement: S15 Fig — (A) Stacked bar plot showing the distribution of carriage and disease isolates between studies. (B) Stacked bar plot showing the distribution of carriage and disease isolates between serotypes. (PNG) [file pcbi.1009389.s017.png]

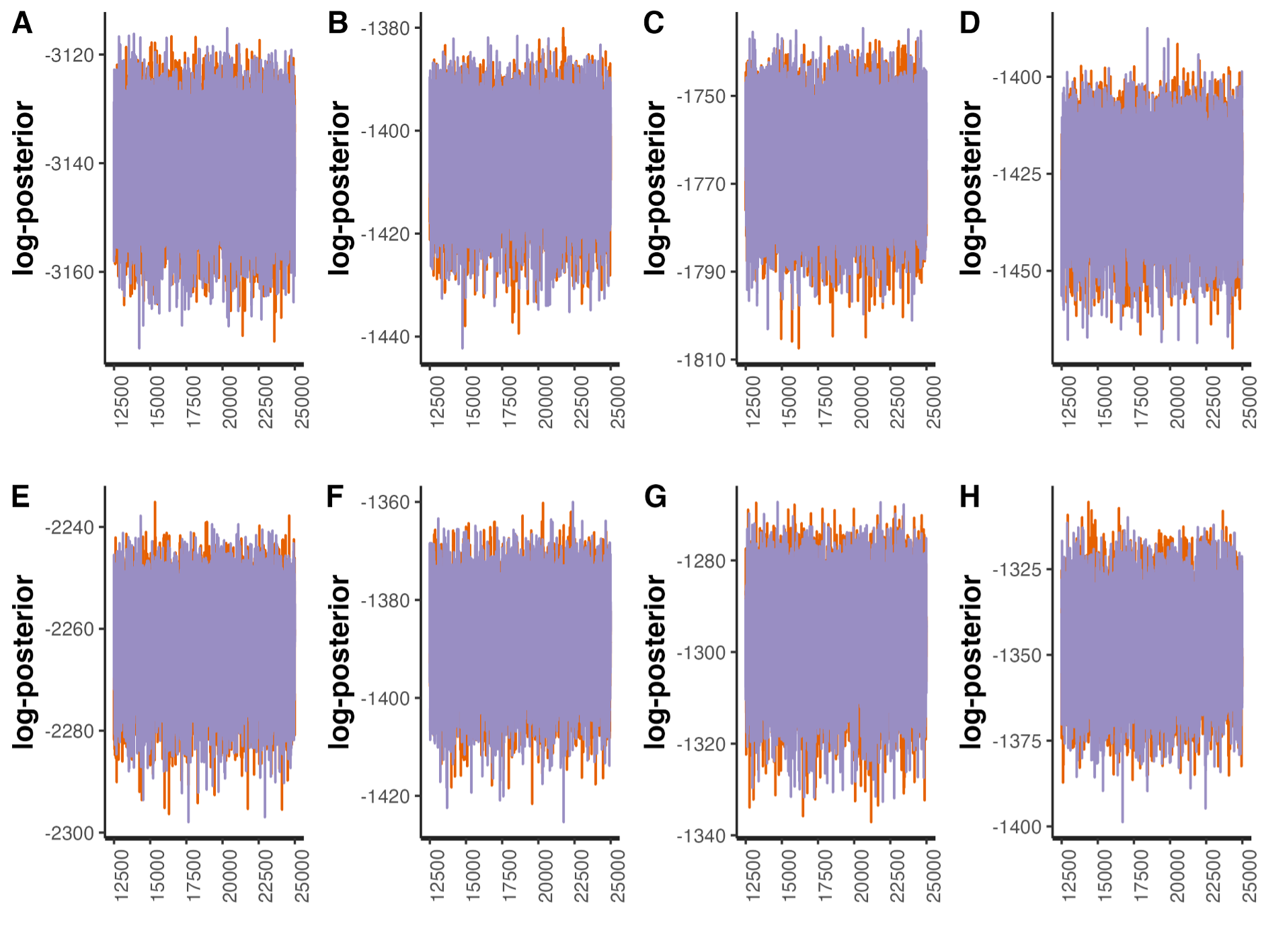

Supplement: S16 Fig — The horizontal axis shows the generation of the MCMC, with values for the two chains shown by orange and purple lines. Each panel corresponds to a different model: (A) null Poisson model; (B) null negative binomial model; (C) type-specific Poisson model; (D) type-specific negative binomial model; (E) study-adjusted Poisson model; (F) study-adjusted negative binomial model; (G) study-adjusted type-specific Poisson model; (H) study-adjusted type-specific negative binomial model. (PNG) [file pcbi.1009389.s018.png]

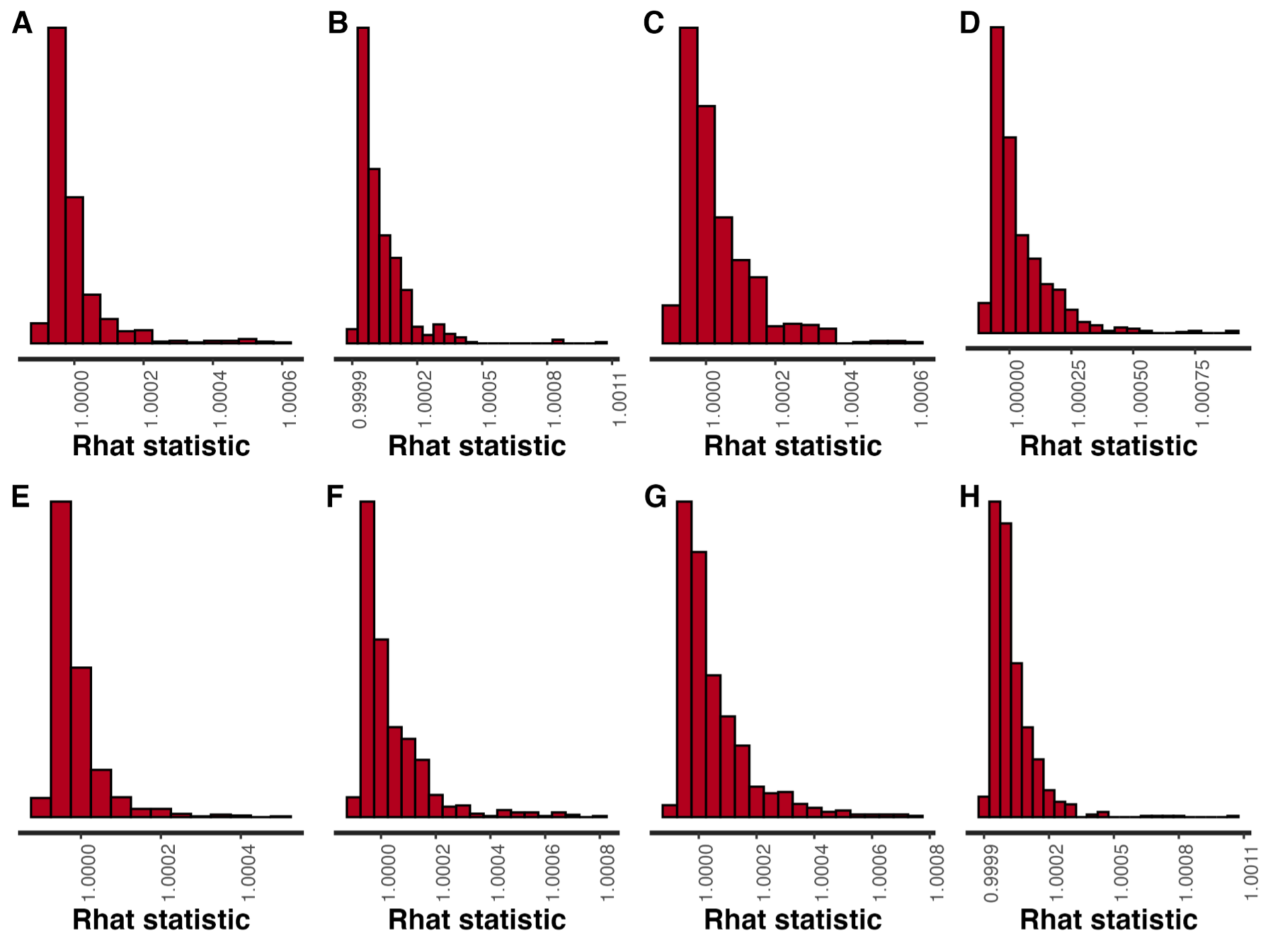

Supplement: S17 Fig — Each panel corresponds to a different model: (A) null Poisson model; (B) null negative binomial model; (C) type-specific Poisson model; (D) type-specific negative binomial model; (E) study-adjusted Poisson model; (F) study-adjusted negative binomial model; (G) study-adjusted type-specific Poisson model; (H) study-adjusted type-specific negative binomial model. (PNG) [file pcbi.1009389.s019.png]

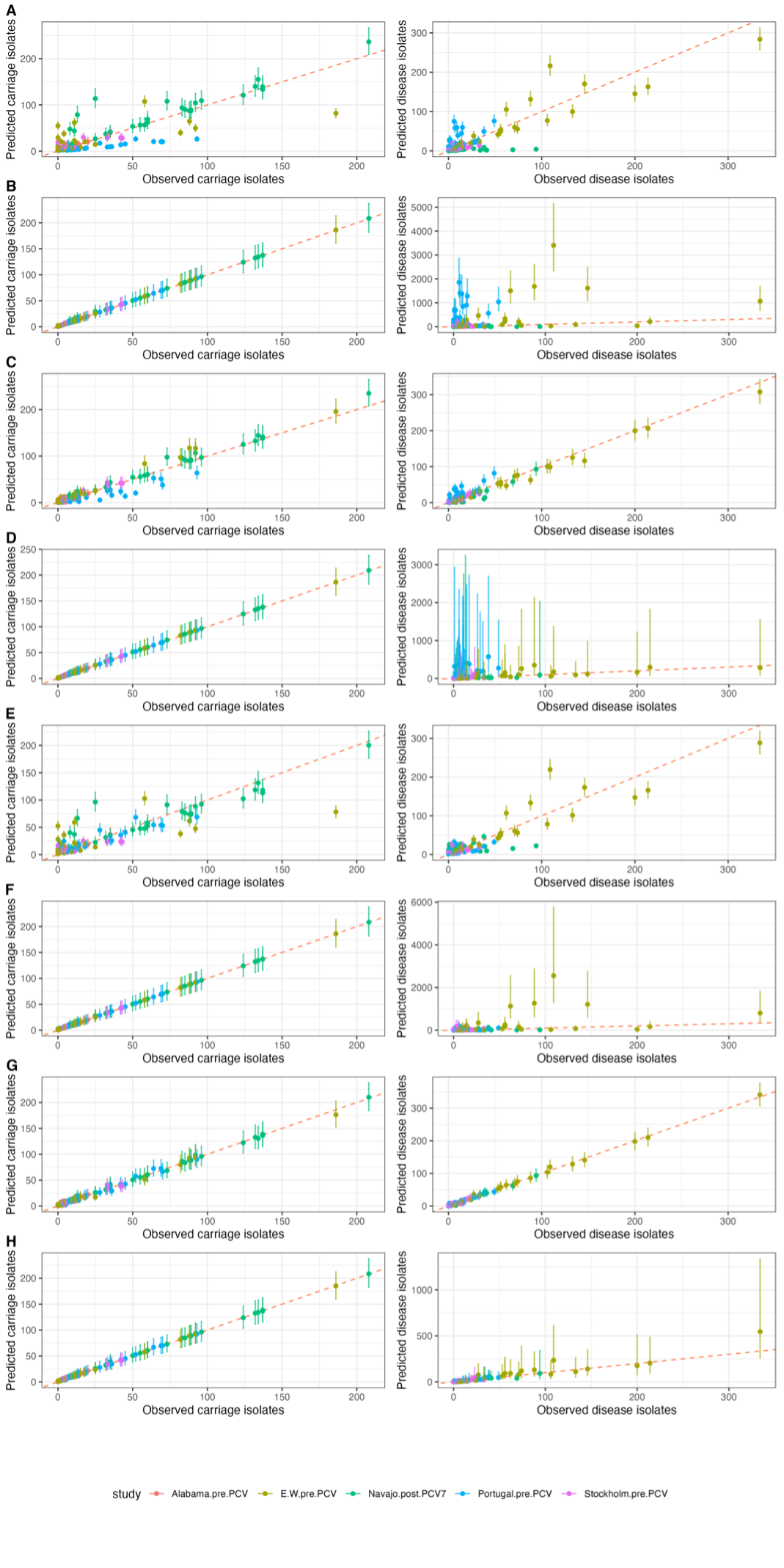

Supplement: S18 Fig — The plots in the left column display data on carriage in children (values of ci,j). The plots in the right column display data on disease in adults (values of di,j). The points are coloured by the study to which they correspond, and represent the observed value on the horizontal axis, and the median predicted value on the vertical axis. The error bars show the 95% credibility intervals. The red dashed line shows the line of identity, corresponding to a perfect match between prediction and observation. Each row corresponds to a different model: (A) null Poisson model; (B) null negative binomial model; (C) type-specific Poisson model; (D) type-specific negative binomial model; (E) study-adjusted Poisson model; (F) study-adjusted negative binomial model; (G) study-adjusted type-specific Poisson model; (H) study-adjusted type-specific negative binomial model. (PNG) [file pcbi.1009389.s020.png]

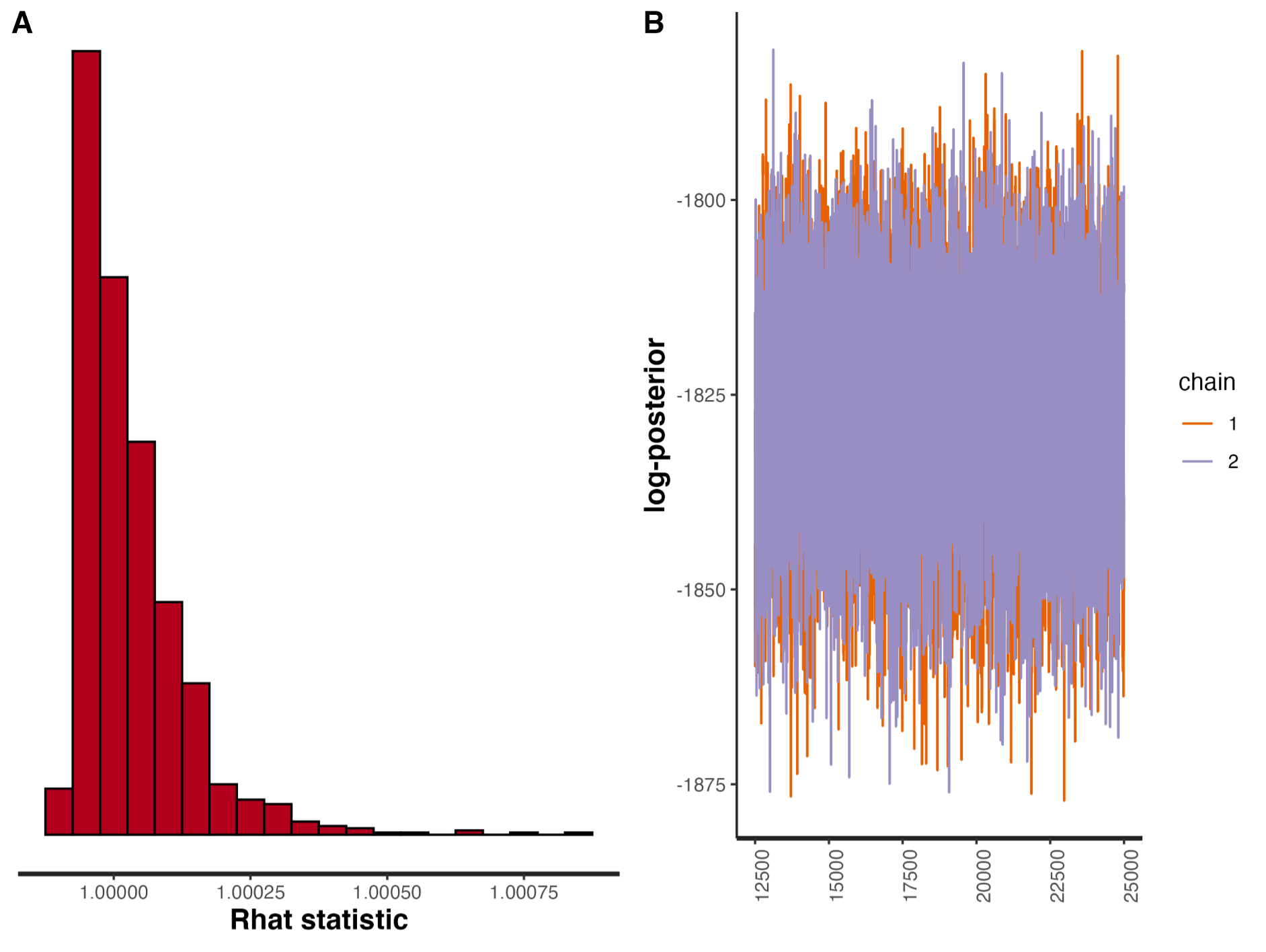

Supplement: S19 Fig — (A) Histogram showing the distribution of R^ values. (B) Post-warmup MCMC traces of the log posterior probability. (PNG) [file pcbi.1009389.s021.png]

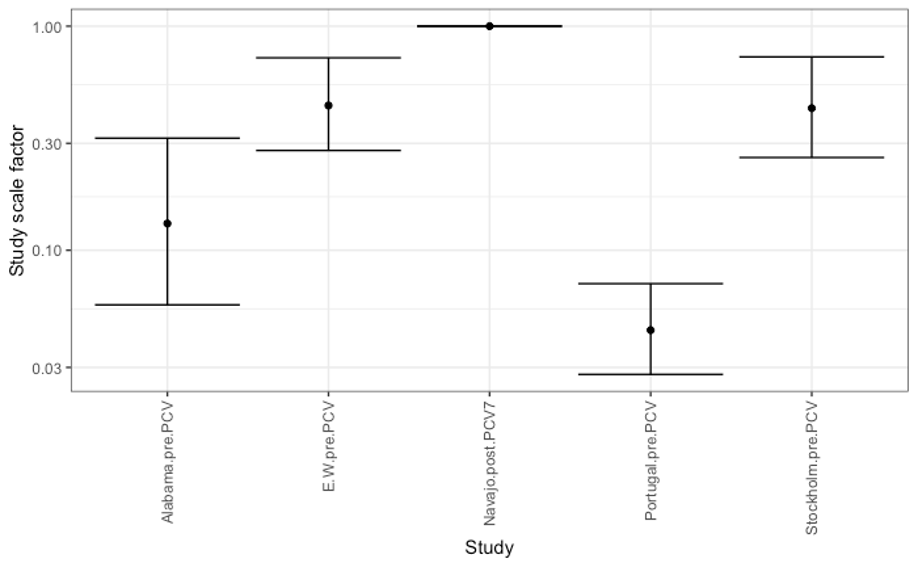

Supplement: S20 Fig — The reference study, for which the value was fixed at one, was the Navajo post-PCV7 dataset, which had the greatest sample size in this meta-analysis (S15 Fig). (PNG) [file pcbi.1009389.s022.png]

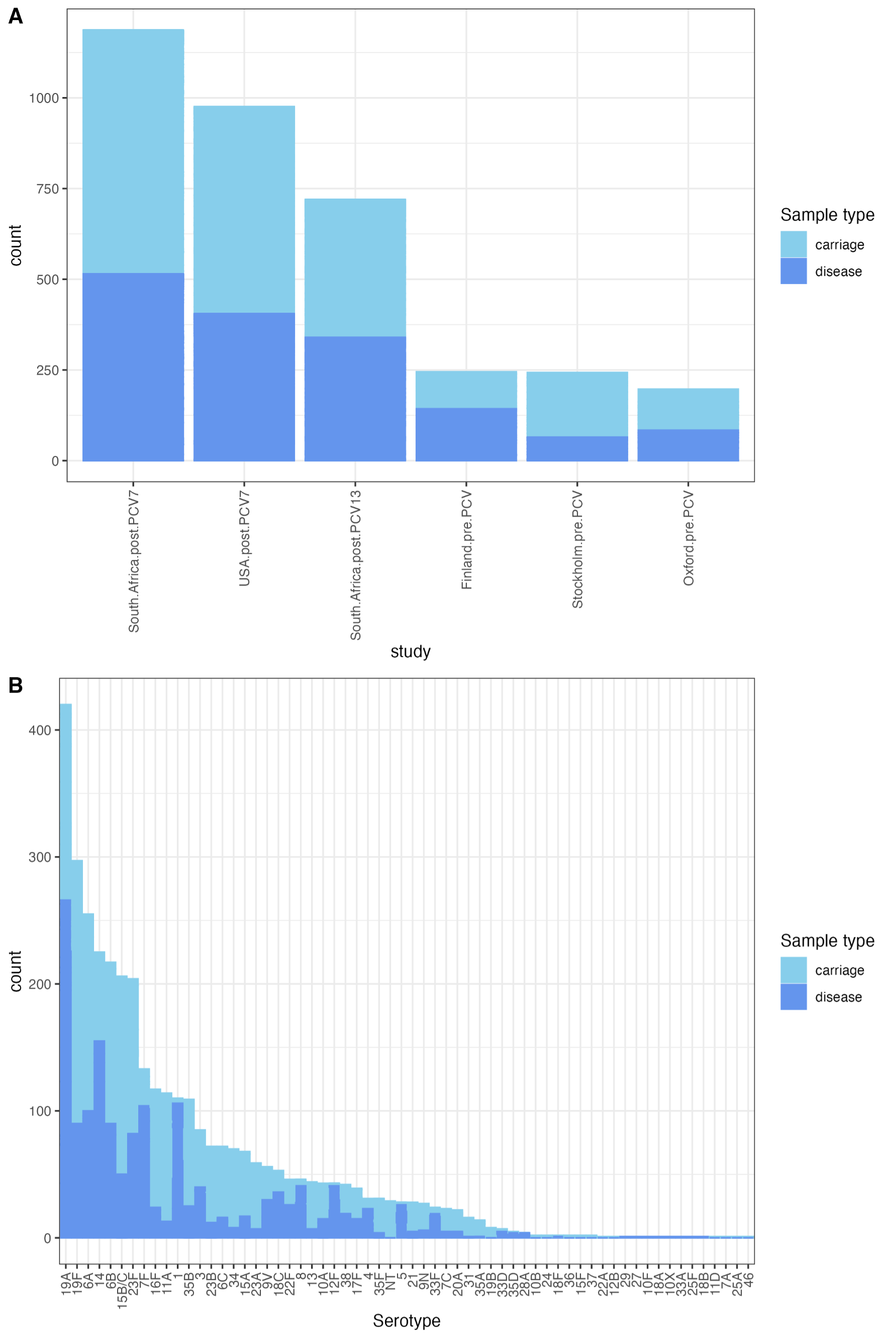

Supplement: S21 Fig — (A) Stacked bar plot showing the distribution of carriage and disease isolates between studies. (B) Stacked bar plot showing the distribution of carriage and disease isolates between serotypes. (PNG) [file pcbi.1009389.s023.png]

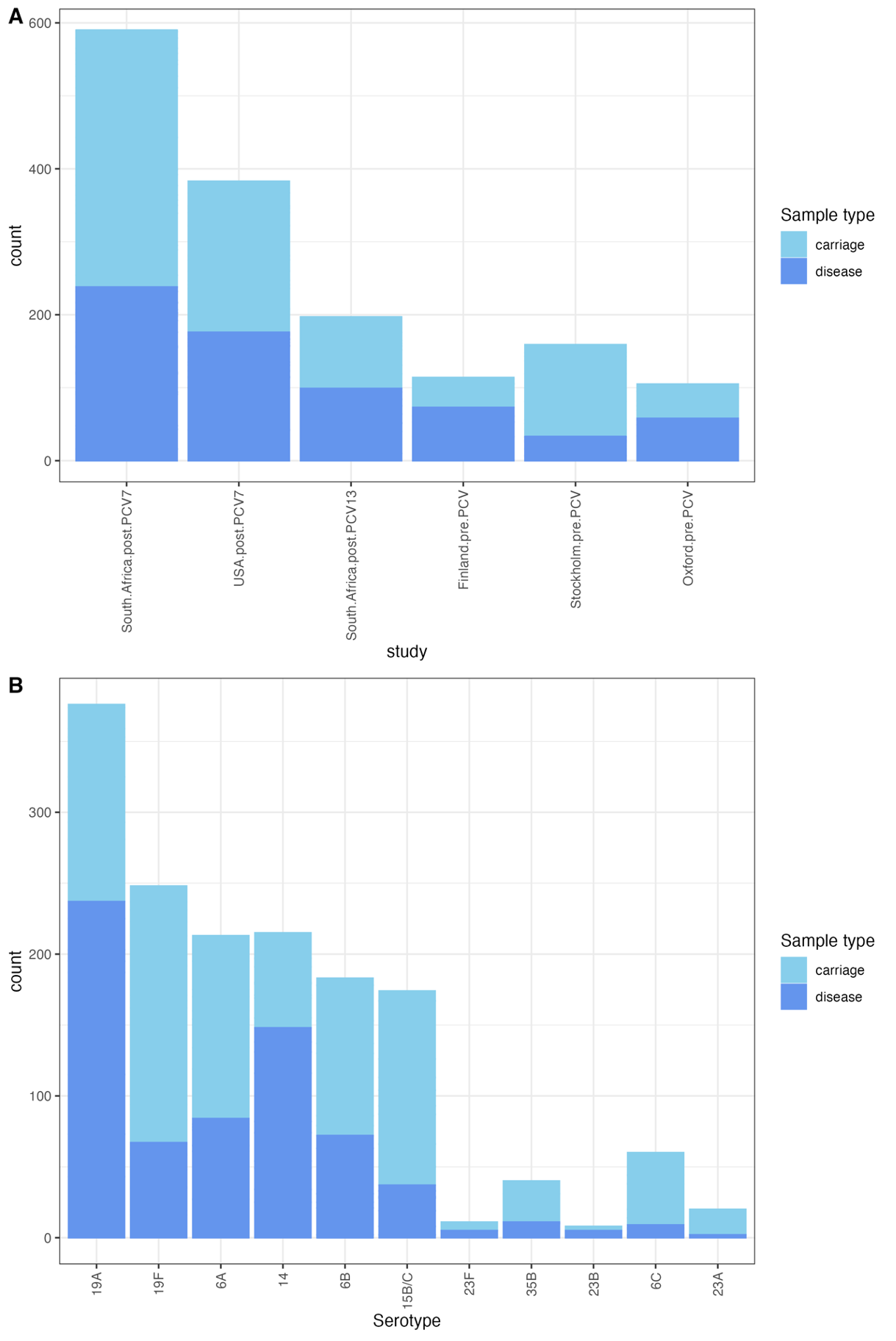

Supplement: S22 Fig — (A) Stacked bar plot showing the distribution of carriage and disease isolates between studies. (B) Stacked bar plot showing the distribution of carriage and disease isolates between serotypes. (PNG) [file pcbi.1009389.s024.png]

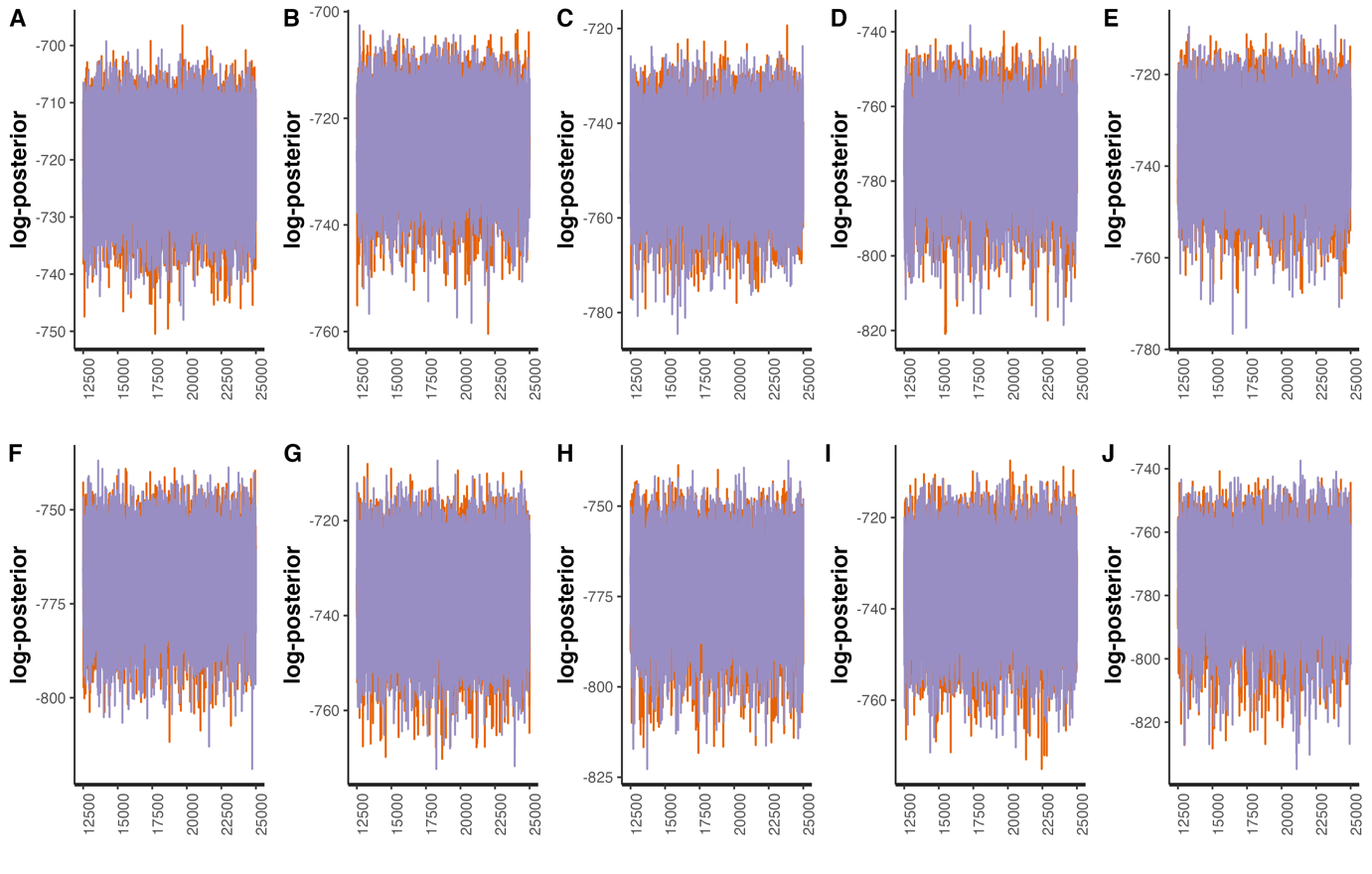

Supplement: S23 Fig — Each panel corresponds to a model with a different method of associating isolates with an invasiveness estimate: (A) serotype-determined, Poisson-distributed invasiveness; (B) serotype-determined, negative binomially-distributed invasiveness; (C) strain-determined, Poisson-distributed invasiveness; (D) strain-determined, negative binomially-distributed invasiveness; (E) serotype-determined, strain-modified Poisson-distributed invasiveness; (F) serotype-determined, strain-modified negative binomially-distributed invasiveness; (G) strain-determined, serotype-modified Poisson-distributed invasiveness; (H) strain-determined, serotype-modified negative binomially-distributed invasiveness; (I) strain- and serotype-determined Poisson-distributed invasiveness; (J) strain- and serotype-determined negative binomially-distributed invasiveness. (PNG) [file pcbi.1009389.s025.png]

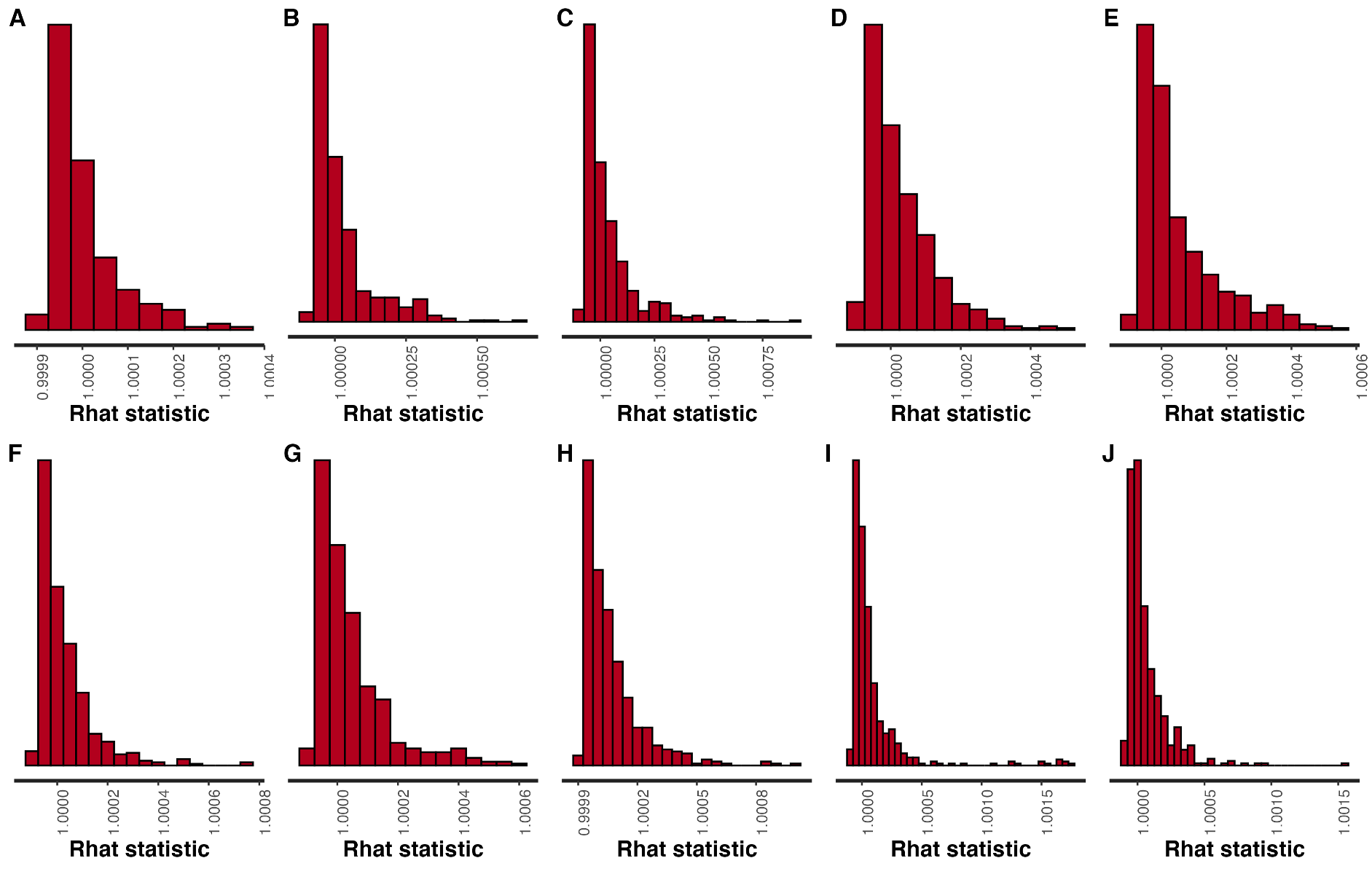

Supplement: S24 Fig — Each panel corresponds to a different model: (A) serotype-determined, Poisson-distributed invasiveness; (B) serotype-determined, negative binomially-distributed invasiveness; (C) strain-determined, Poisson-distributed invasiveness; (D) strain-determined, negative binomially-distributed invasiveness; (E) serotype-determined, strain-modified Poisson-distributed invasiveness; (F) serotype-determined, strain-modified negative binomially-distributed invasiveness; (G) strain-determined, serotype-modified Poisson-distributed invasiveness; (H) strain-determined, serotype-modified negative binomially-distributed invasiveness; (I) strain- and serotype-determined Poisson-distributed invasiveness; (J) strain- and serotype-determined negative binomially-distributed invasiveness. (PNG) [file pcbi.1009389.s026.png]

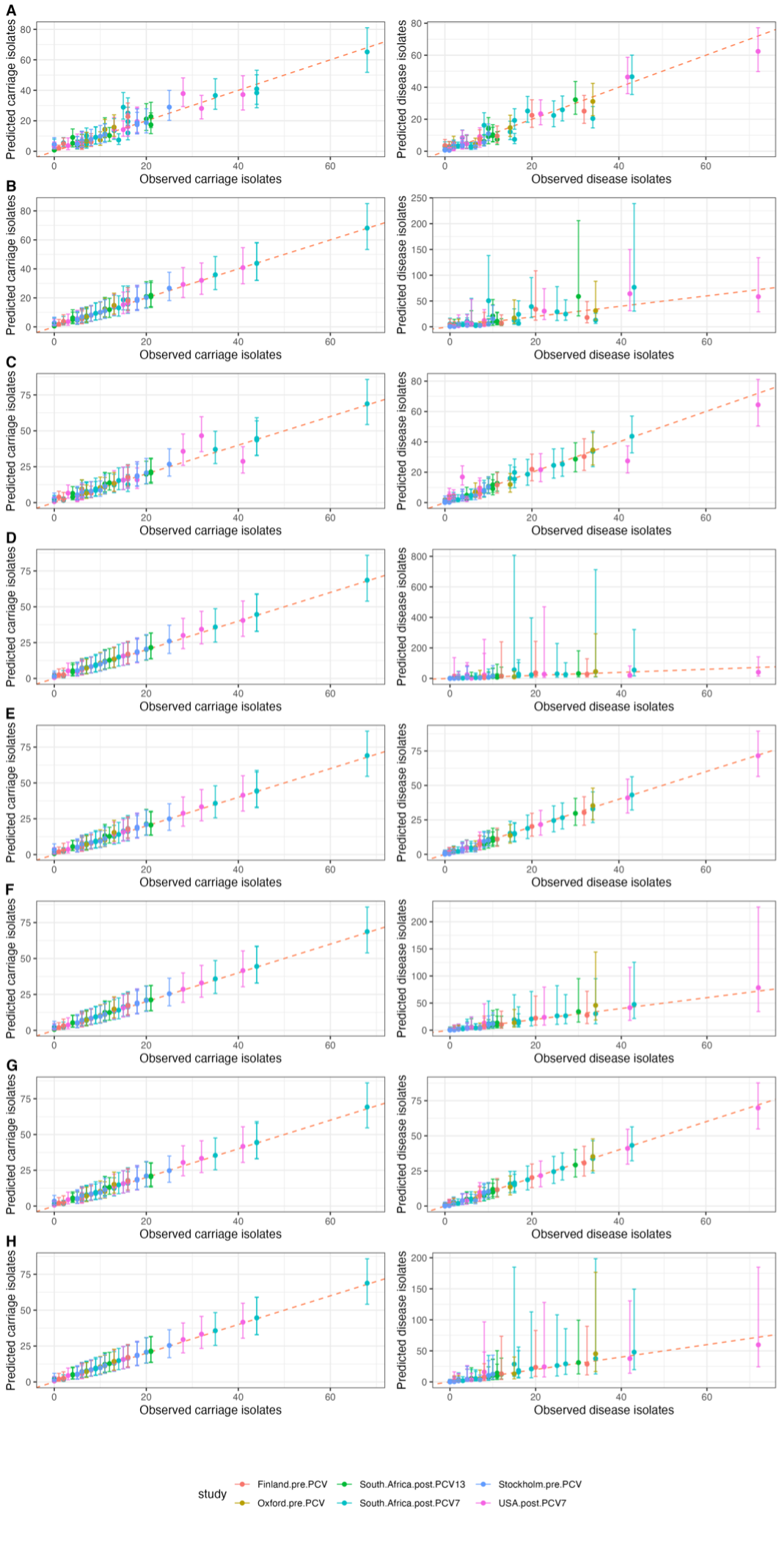

Supplement: S25 Fig — The points are coloured by the study to which they correspond, and represent the observed value on the horizontal axis, and the median predicted values on the vertical axis. The error bars show the 95% credibility intervals. The red dashed line shows the line of identity, corresponding to a perfect match between prediction and observation. The left column shows the correspondence for carriage data (values of ci,j,k), and the right column shows the correspondence for disease isolates (values of di,j,k). Each row corresponds to a different model: (A) serotype-determined, Poisson-distributed invasiveness; (B) serotype-determined, negative binomially-distributed invasiveness; (C) strain-determined, Poisson-distributed invasiveness; (D) strain-determined, negative binomially-distributed invasiveness; (E) serotype-determined, strain-modified Poisson-distributed invasiveness; (F) serotype-determined, strain-modified negative binomially-distributed invasiveness; (G) strain-determined, serotype-modified Poisson-distributed invasiveness; (H) strain-determined, serotype-modified negative binomially-distributed invasiveness; (I) strain- and serotype-determined Poisson-distributed invasiveness; (J) strain- and serotype-determined negative binomially-distributed invasiveness. (PNG) [file pcbi.1009389.s027.png]

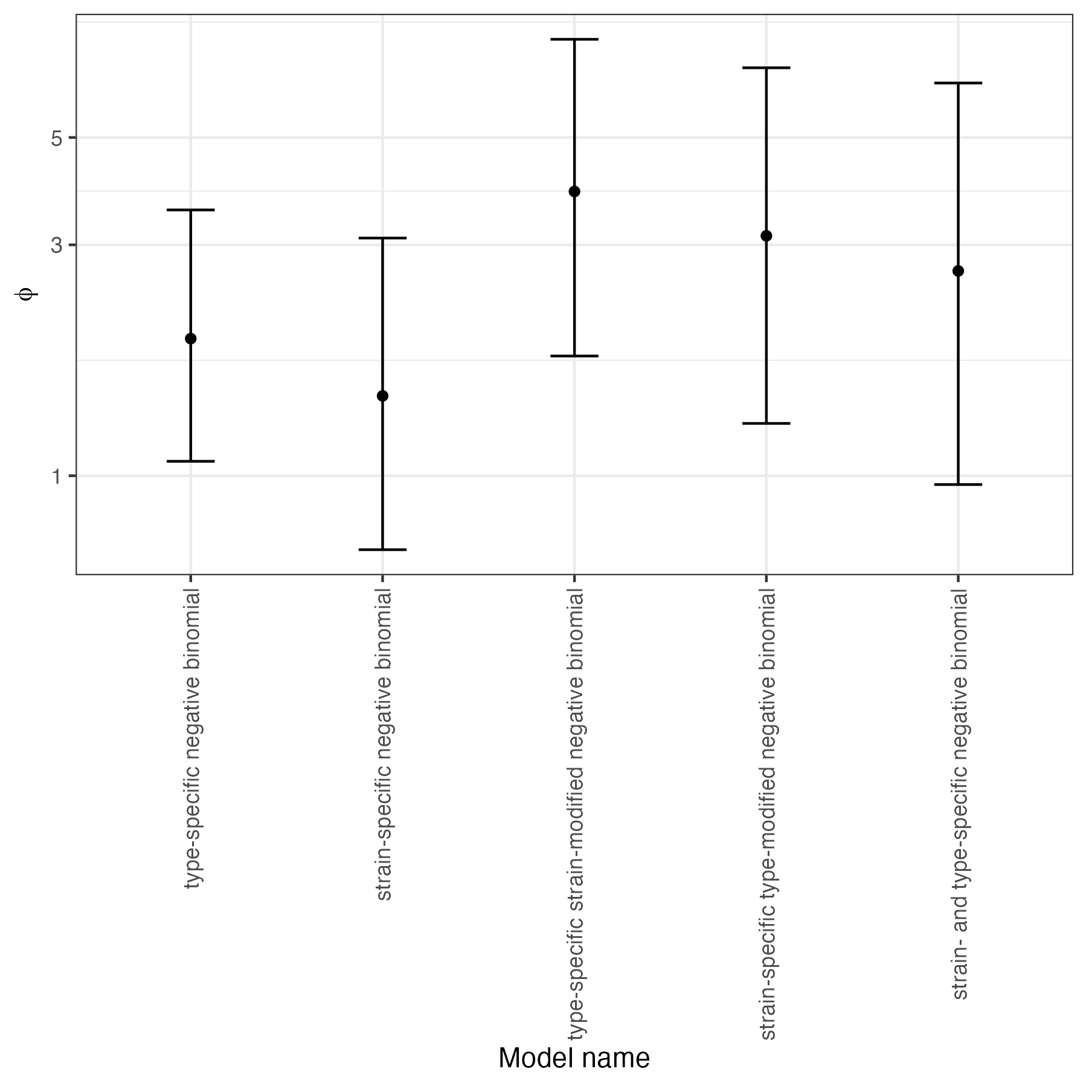

Supplement: S26 Fig — The points represent the median estimates from the MCMCs, and the error bars show the 95% credibility interval. (PNG) [file pcbi.1009389.s028.png]

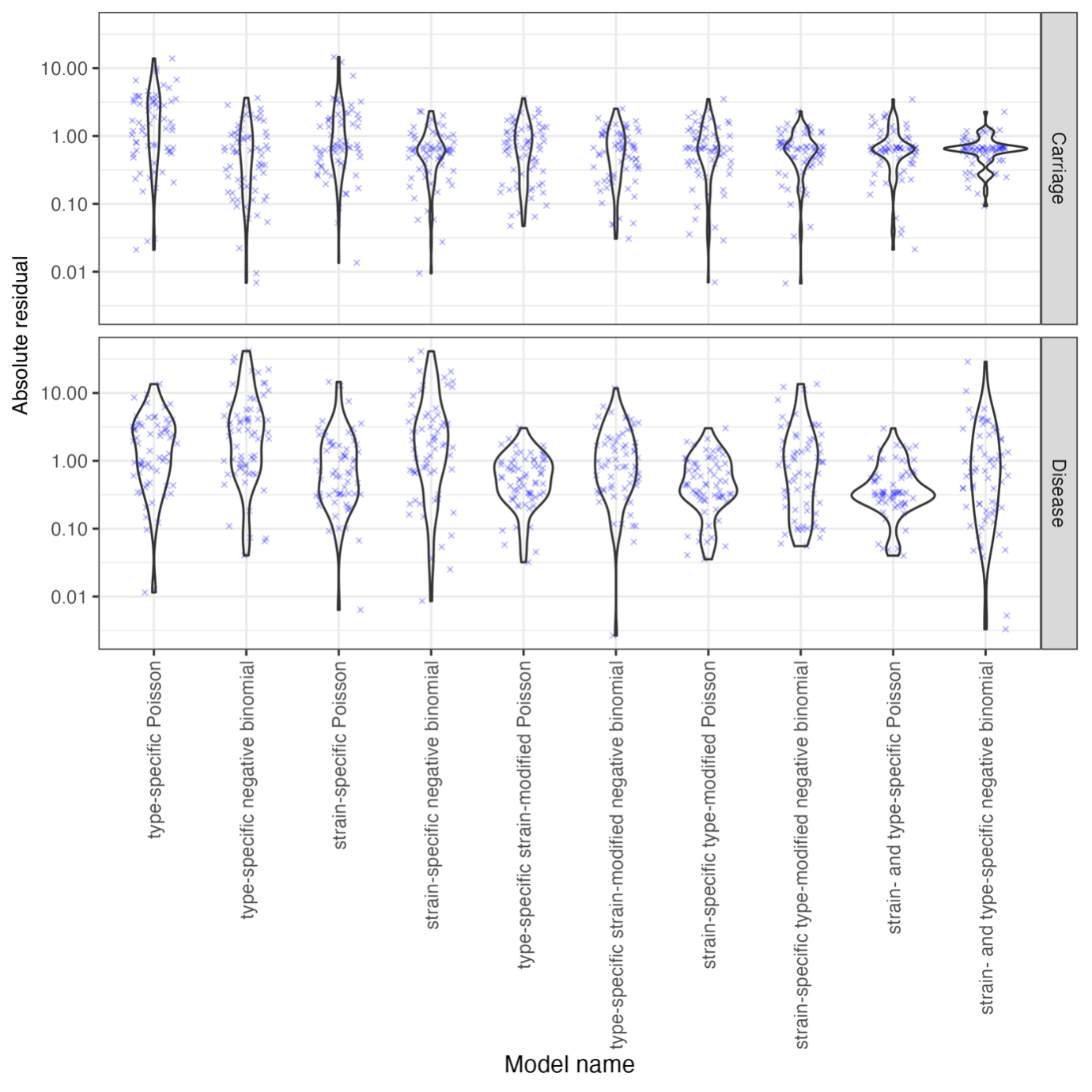

Supplement: S27 Fig — Blue crosses represent the individual observations. (PNG) [file pcbi.1009389.s029.png]

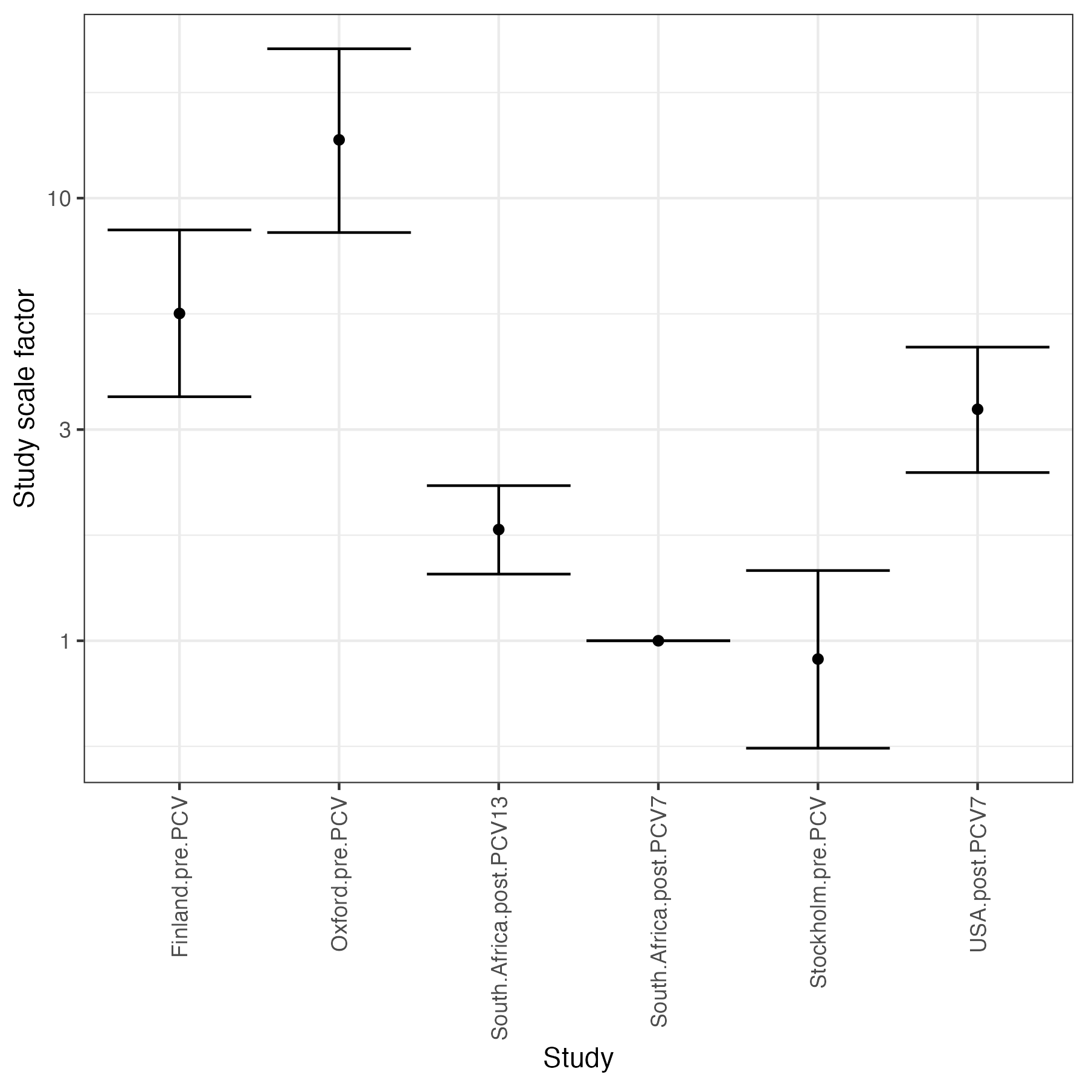

Supplement: S28 Fig — The reference study, for which the value was fixed at one, was the South Africa post-PCV7 dataset, which had the greatest sample size in this meta-analysis, and was associated with a known carriage sample size (S2 Text). (PNG) [file pcbi.1009389.s030.png]

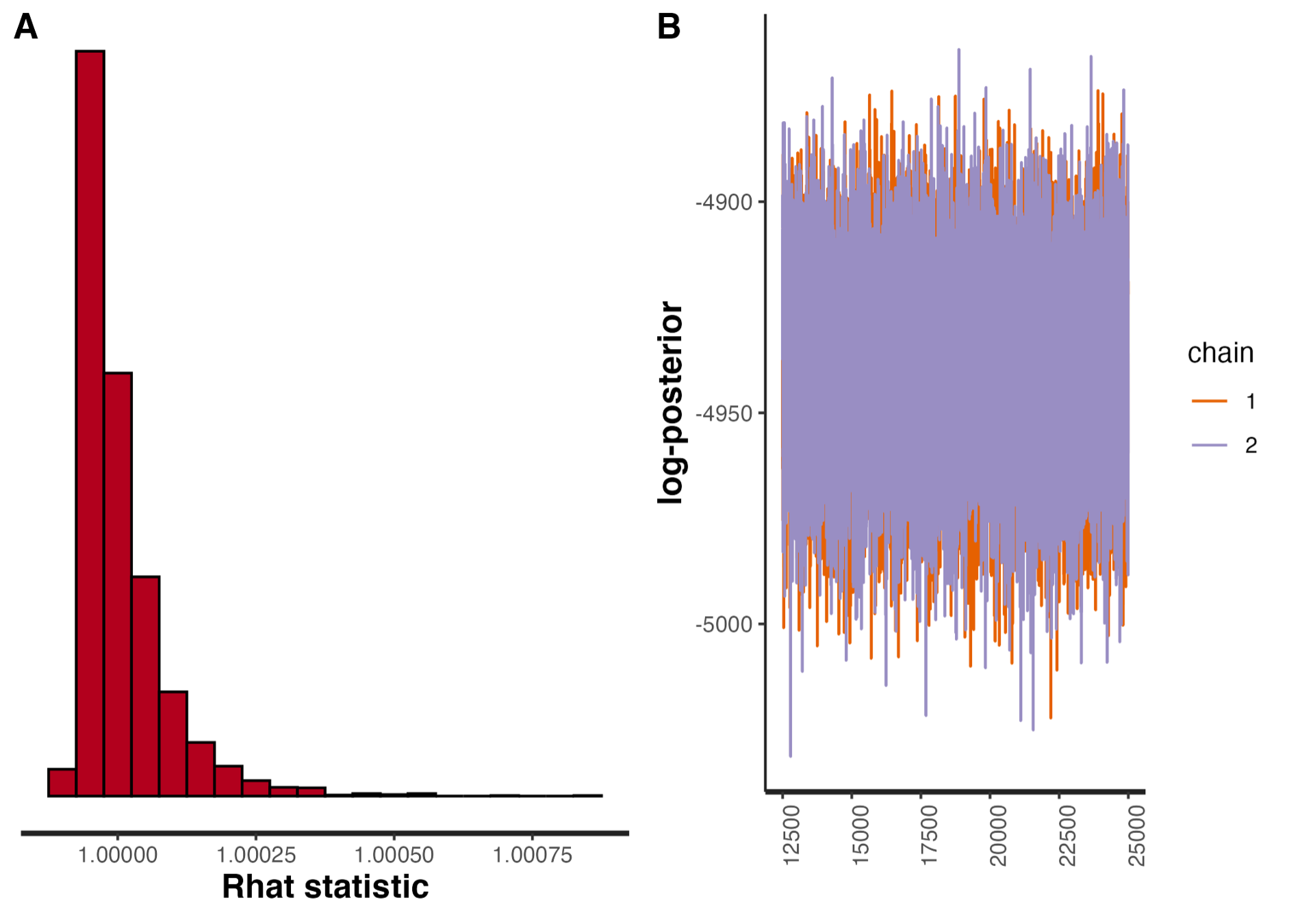

Supplement: S29 Fig — (A) Histogram showing the distribution of R^ values. (B) Post-warmup MCMC traces of the log posterior probability. (PNG) [file pcbi.1009389.s031.png]

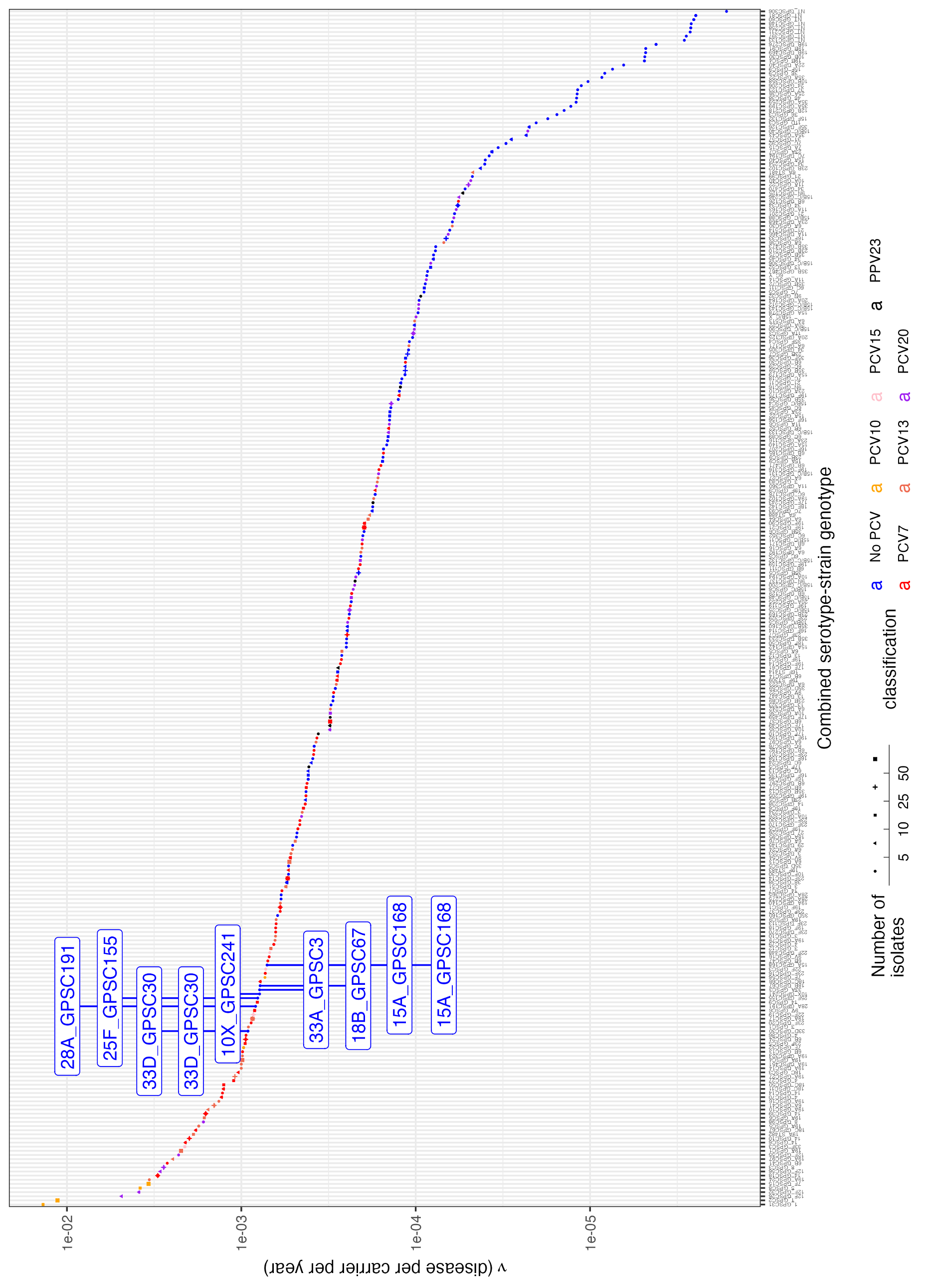

Supplement: S30 Fig — The shape of each point represents the number of isolates of each combination observed across carriage and disease samples. The serotype-strain combinations with the highest combined invasiveness that are not targeted by current PCV designs are labelled. (PNG) [file pcbi.1009389.s032.png]

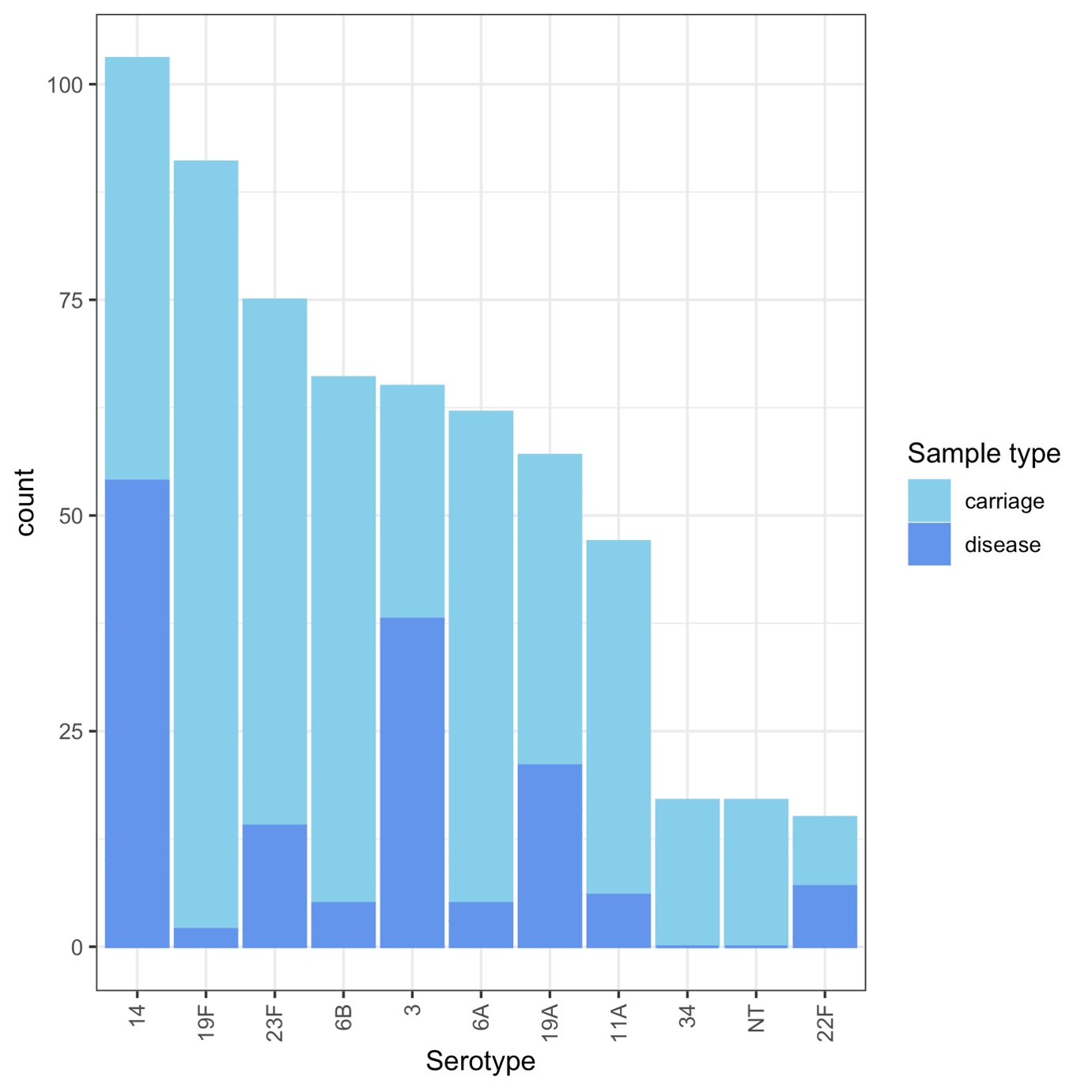

Supplement: S31 Fig — (PNG) [file pcbi.1009389.s033.png]

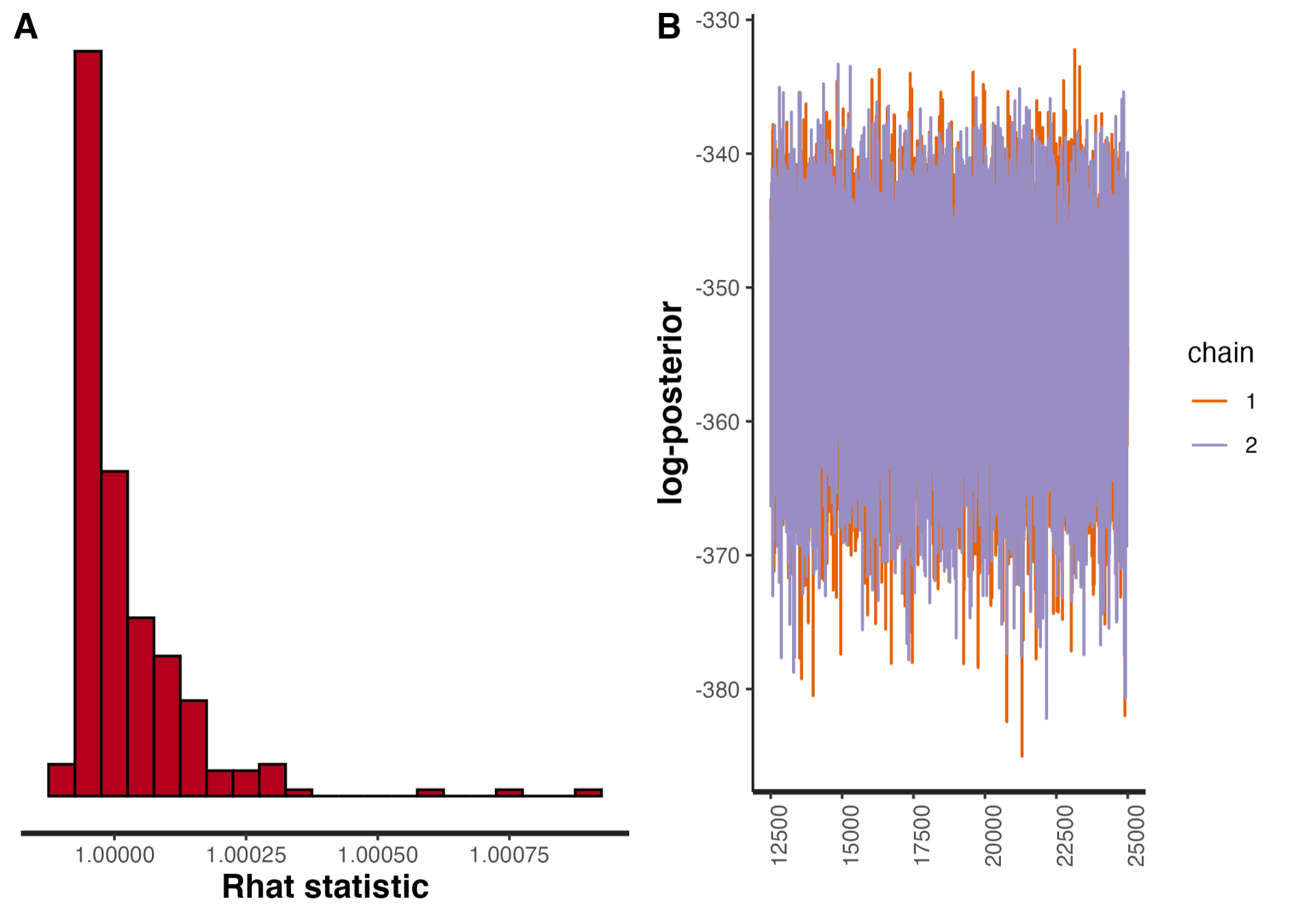

Supplement: S32 Fig — (A) Histogram showing the distribution of R^ values. (B) Post-warmup MCMC traces of the log posterior probability. (PNG) [file pcbi.1009389.s034.png]

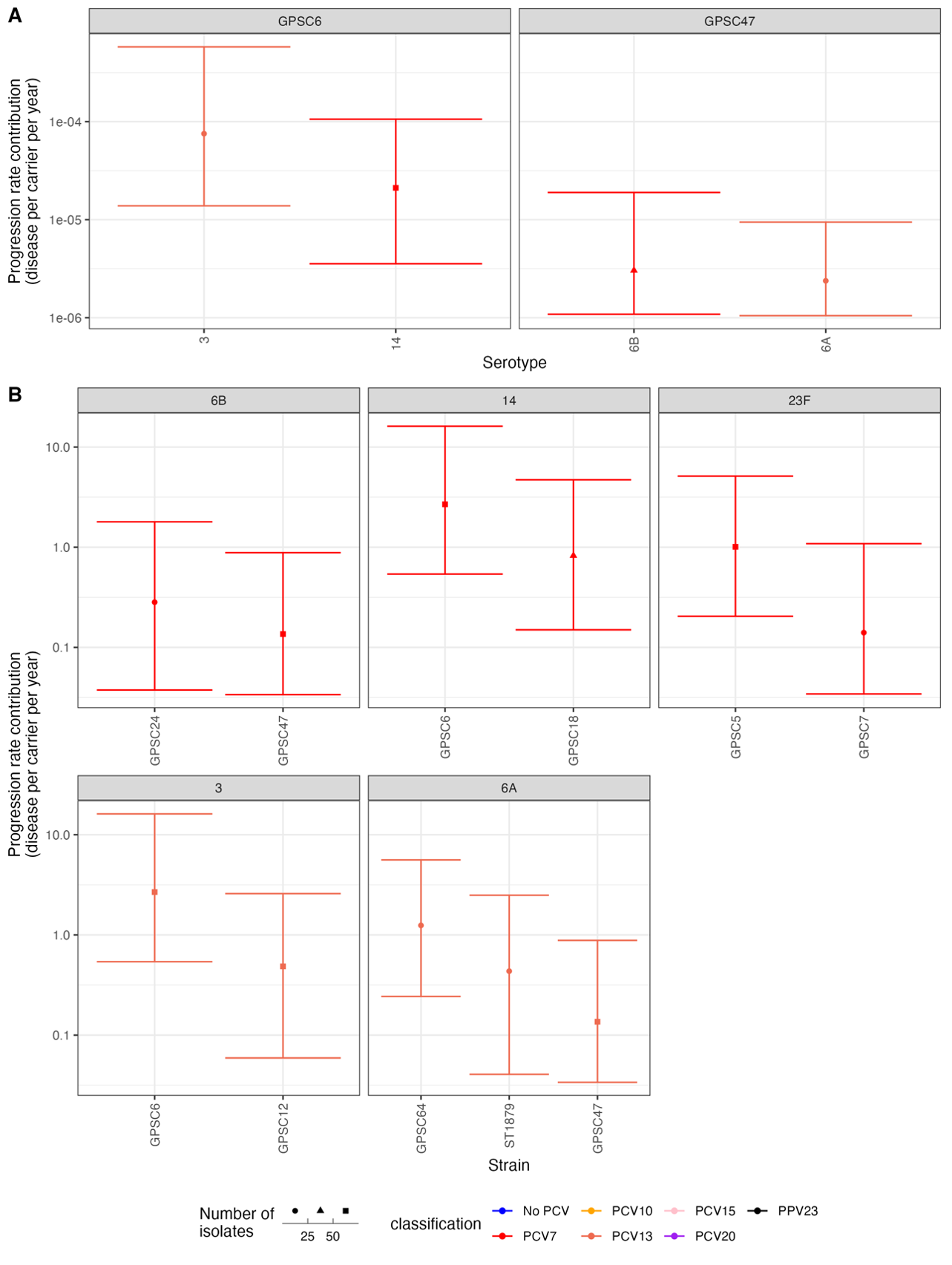

Supplement: S33 Fig — Points represent the median estimates, and are coloured by the vaccine formulations in which the corresponding serotype is present. The error bars represent 95% credibility intervals. The shape of the point represents the sample size on which the estimate is based. (A) Estimates of the invasiveness associated with serotypes, arranged by the strains in which they are found. Only strains expressing multiple serotypes are displayed. (B) Estimates of the coefficient by which strains modify the invasiveness of their expressed serotype, arranged by the serotypes with which they are associated. Only serotypes associated with multiple strains are displayed. (PNG) [file pcbi.1009389.s035.png]
