# Supplementary material for: Analysing pneumococcal invasiveness using Bayesian models of pathogen progression rates
Source: PLoS Comput Biol. 2022 Feb 17;18(2):e1009389. doi: 10.1371/journal.pcbi.1009389 (PMC8901055; doi:10.1371/journal.pcbi.1009389)
Supplement: S1 Table — (DOCX) [file pcbi.1009389.s036.docx]

| **Population** | **Vaccine period** | **Carriage study time interval** | **Carriage isolate source** | **Disease isolate source** | **No. of swabs** | **Population of children** | **No. disease isolates from children** | **Population of adults** | **No. disease isolates from adults** | **References** |
| --- | --- | --- | --- | --- | --- | --- | --- | --- | --- | --- |
| Alabama | Pre-PCV | July 1975 – December 1978 | Unvaccinated sick and healthy children <18 years old from Alabama | Unvaccinated children with IPD <18 years old from Alabama and unvaccinated adults hospitalised with pneumonia or IPD in Alabama | 827 | 19,316 | 114 | 232,373 | 86 | [1,2] |
| Atlanta | Pre-PCV | January – December 1995 | Unvaccinated children with recent URI in Atlanta <5 years old | Unvaccinated children <5 years old with IPD in Atlanta | 231 | 204,680 | 202 | - | - | [3] |
| Bogota | Pre-PCV | May 2005 – November 2006 | Healthy unvaccinated children <18 months old in Bogota | IPD in children <2 years old in Bogota | 197 | 357,200 | 353 | - | - | [4] |
| Caracas | Pre-PCV | December 2006 – January 2008 | Unvaccinated healthy children <6 years old in Caracas | Unvaccinated children with IPD <6 years old in Caracas | 1,004 | 146,125 | 36 | - | - | [5,6] |
| Czech | Pre-PCV | 1996 – 2005 | Unvaccinated healthy children 3 - 5 years old across the Czech Republic | Unvaccinated children <6 years old with IPD across the Czech Republic | 425 | 478,177 | 138 | - | - | [7,8] |
| England & Wales | Pre-PCV | July 1996 – June 2006 | Unvaccinated healthy children <5 years old in Hertfordshire | Unvaccinated children <5 years old across and predominantly unvaccinated individuals >4 years old across England & Wales | 3,752 | 3,091,000 | 461 | 48,702,414 | 1,876 | [9] |
| Goroka | Pre-PCV | 1981 – 1987 | Unvaccinated children attending clinics near Goroka Town | Unvaccinated children with IPD in Goroka hospital | 2,844 | 96,207 | 56 | - | - | [10,11] |
| Morocco | Pre-PCV | November 2010 – December 2011 | Unvaccinated healthy children in Rabat | Children hospitalised with severe pneumonia in Rabat <5 years old | 200 | 212,566 | 118 | - | - | [12,13] |
| Netherlands | Pre-PCV | June 2004 – May 2006 | Unvaccinated healthy children <2 years old in Noord-Holland, Zuid-Holland and Utrecht | Children <5 years with IPD across Netherlands | 321 | 250,924 | 100 | - | - | [14,15] |
| Ontario | Pre-PCV | 1995 | Unvaccinated healthy children primarily <4 years old in Toronto | Unvaccinated children <18 years old with IPD in Toronto and surrounding area | 1,139 | 580,507 | 89 | - | - | [16] |
| Portugal | Pre-PCV | January 2001 – December 2003 | Unvaccinated healthy children <7 years old in Lisbon and Oeiras | Unvaccinated children (<18 years) and adults (>18 years) across Portugal | 1,170 | 2,071,223 | 90 | 8,284,894 | 378 | [17,18] |
| Stockholm | Pre-PCV | 1997 | Unvaccinated healthy children <7 years old in Stockholm | Unvaccinated adults (and a small number of children) hospitalised with IPD in Stockholm | 611 | - | - | 2,004,152 | 273 | [19,20] |
| Atlanta | Post-PCV7 | June 2008 – May 2009 | PCV7-vaccinated sick Georgia-resident children <5 years old | PCV7-vaccinated children <5 years old with IPD in Atlanta | 451 | 298,831 | 47 | - | - | [3] |
| Barcelona | Post-PCV7 | 2007 – 2011 | PCV7-vaccinated healthy children <7 years old in Barcelona | PCV7-vaccinated children <7 years old with IPD in Barcelona | 209 | 228,000 | 159 | - | - | [21] |
| Bogota | Post-PCV7 | June – November 2011 | PCV7-vaccinated healthy children <18 months old in Bogota | IPD in children <2 years old in Bogota | 246 | 357,200 | 91 | - | - | [4] |
| France | Post-PCV7 | January 2008 – December 2009 | PCV7-vaccinated healthy children <2 years old across France | PCV7-vaccinated children with IPD <2 years old across France | 1,212 | 838,866 | 388 | - | - | [22,23] |
| Massachusetts | Post-PCV7 | 2001 – April 2009 | PCV7-vaccinated children visiting physicians <7 years old across Massachusetts | PCV7-vaccinated children <7 years with IPD old across Massachusetts | 2,969 | 820,000 | 206 | - |  | [24] |
| Navajo | Post-PCV7 | March 2006 - March 2008 | PCV7-vaccinated Navajo or White Mountain Apache native American children <9 years old | Active IPD surveillance of PCV7-vaccinated Navajo or White Mountain Apache native American children <7 years old and people at least 18 years old | 6,541 | 65,048 | 132 | 201,553 | 514 | [25–27] |
| Netherlands | Post-PCV7 | June 2008 – May 2012 | Vaccinated healthy children <2 years old in Noord-Holland, Zuid-Holland and Utrecht | IPD in children (<5 years) across Netherlands | 660 | 232,251 | 73 | - | - | [14,15] |
| France | Post-PCV13 | January 2012 – December 2013 | PCV13-vaccinated healthy children <2 years old across France | PCV13-vaccinated children with IPD <2 years old across France | 1,212 | 842,076 | 181 | - | - | [22,23] |
| Netherlands | Post-PCV10 | June 2012 – May 2016 | Vaccinated healthy children <2 years old in Noord-Holland, Zuid-Holland and Utrecht | IPD in children (<5 years) across Netherlands | 659 | 222,671 | 47 | - | - | [14,15] |

**References**

1. Gibson C. Population of the 100 Largest Cities and Other Urban Places In The United States: 1790 to 1990. 1998. Available: https://www.census.gov/library/working-papers/1998/demo/POP-twps0027.html

2. Gray BM, Converse GM, Dillon HC. Serotypes of *Streptococcus pneumoniae* causing disease. J Infect Dis. 1979;140: 979–83. doi:10.1093/infdis/140.6.979

3. Sharma D, Baughman W, Holst A, Thomas S, Jackson D, Da Gloria Carvalho M, et al. Pneumococcal carriage and invasive disease in children before introduction of the 13-valent conjugate vaccine: Comparison with the era before 7-valent conjugate vaccine. Pediatr Infect Dis J. 2013;32: e45-53. doi:10.1097/INF.0b013e3182788fdd

4. Parra EL, De La Hoz F, Díaz PL, Sanabria O, Realpe ME, Moreno J. Changes in *Streptococcus pneumoniae* serotype distribution in invasive disease and nasopharyngeal carriage after the heptavalent pneumococcal conjugate vaccine introduction in Bogotá, Colombia. Vaccine. 2013;31: 4033–8. doi:10.1016/j.vaccine.2013.04.074

5. Instituto Nacional de Estadistica Venezuela. Censo Nacional de Poblacion y Vivienda. 2011. Available: http://www.redatam.ine.gob.ve/Censo2011/index.html

6. Rivera-Olivero IA, Del Nogal B, Sisco MC, Bogaert D, Hermans PWM, De Waard JH. Carriage and invasive isolates of *Streptococcus pneumoniae* in Caracas, Venezuela: The relative invasiveness of serotypes and vaccine coverage. Eur J Clin Microbiol Infect Dis. 2011;30: 1489–95. doi:10.1007/s10096-011-1247-5

7. Czech Statistical Office. Age Distribution of the Population of the Czech Republic - 2005. 2005. Available: https://www.czso.cz/csu/czso/4003-06-2005-01n

8. Zemlickova H, Jakubu V, Urbaskova P, Motlova J, Musilek M, Adamkova V. Serotype-specific invasive disease potential of *Streptococcus pneumoniae* in Czech children. J Med Microbiol. 2010;59: 1079–83. doi:10.1099/jmm.0.018390-0

9. Trotter CL, Waight P, Andrews NJ, Slack M, Efstratiou A, George R, et al. Epidemiology of invasive pneumococcal disease in the pre-conjugate vaccine era: England and Wales, 1996-2006. J Infect. 2010;60: 200–8. doi:10.1016/j.jinf.2009.12.008

10. Papua New Guinea National Statistical Office. National Population and Housing Census Ward Population Profile: Highlands Region. 2011. Available: https://www.nso.gov.pg/index.php/population-and-social/other-indicators#highlands-region

11. Smith T, Lehmann D, Montgomery J, Gratten M, Riley ID, Alpers MP. Acquisition and invasiveness of different serotypes of *Streptococcus pneumoniae* in young children. Epidemiol Infect. 1993;111: 27–39. doi:10.1017/S0950268800056648

12. Haut-Commissariat au Plan. Annuaire statistique de la région Rabat-Salé-Zemmour-Zaër, 2010. 2012. Available: https://www.hcp.ma/downloads/Annuaires-statistiques-regionaux_t11956.html

13. Jroundi I, Mahraoui C, Benmessaoud R, Moraleda C, Munoz Almagro C, Seffar M, et al. *Streptococcus pneumoniae* carriage among healthy and sick pediatric patients before the generalized implementation of the 13-valent pneumococcal vaccine in Morocco from 2010 to 2011. J Infect Public Health. 2017;10: 165–170. doi:10.1016/j.jiph.2016.02.012

14. United Nations Department of Economic and Social Affairs. Population by age groups - both sexes. 2020. Available: https://population.un.org/wpp/Download/Standard/Population/

15. Vissers M, Wijmenga-Monsuur AJ, Knol MJ, Badoux P, van Houten MA, van der Ende A, et al. Increased carriage of non-vaccine serotypes with low invasive disease potential four years after switching to the 10-valent pneumococcal conjugate vaccine in The Netherlands. PLoS One. 2018;13: 1–15. doi:10.1371/journal.pone.0194823

16. Statistics Canada. Data tables, 1996. 1996. Available: https://www.statcan.gc.ca/

17. Instituto Nacionalde Estadistica Portugal. Censos 2001. 2002.

18. Sá-Leao R, Pinto F, Aguiar S, Nunes S, Carriço JAJA, Frazao N, et al. Analysis of invasiveness of pneumococcal serotypes and clones circulating in Portugal before widespread use of conjugate vaccines reveals heterogeneous behavior of clones expressing the same serotype. J Clin Microbiol. 2011;49: 1369–75. doi:10.1128/jcm.01763-10

19. Statistics Sweden. Population by region, marital status, age and sex. Year 1968 - 2018. Available: http://www.statistikdatabasen.scb.se/pxweb/en/ssd/START__BE__BE0101__BE0101A/BefolkningNy/?rxid=e550e75b-4cef-4e77-b5e5-83adcc09c97e

20. Sandgren A, Christensson B, Samuelsson A, Olsson‐Liljequist B, Sjöström K, Kronvall G, et al. Effect of Clonal and Serotype‐Specific Properties on the Invasive Capacity of *Streptococcus pneumoniae*. J Infect Dis. 2004;189: 785–96. doi:10.1086/381686

21. del Amo E, Brotons P, Monsonis M, Triviño M, Iñigo M, Selva L, et al. High invasiveness of pneumococcal serotypes included in the new generation of conjugate vaccines. Clin Microbiol Infect. 2014;20: 684–9. doi:10.1111/1469-0691.12422

22. Institut National de la Statistique et des Etudes Economiques. Age pyramid 2020 – France and metropolitan France. 2008.

23. Varon E, Cohen R, Béchet S, Doit C, Levy C. Invasive disease potential of pneumococci before and after the 13-valent pneumococcal conjugate vaccine implementation in children. Vaccine. 2015;33: 6178–85. doi:10.1016/j.vaccine.2015.10.015

24. Yildirim I, Hanage WP, Lipsitch M, Shea KM, Stevenson A, Finkelstein J, et al. Serotype specific invasive capacity and persistent reduction in invasive pneumococcal disease. Vaccine. 2010/10/30. 2010;29: 283–288. doi:S0264-410X(10)01502-1 [pii]10.1016/j.vaccine.2010.10.032 [doi]

25. Weinberger DM, Bruden DT, Grant LR, Lipsitch M, O’Brien KL, Pelton SI, et al. Using pneumococcal carriage data to monitor postvaccination changes in invasive disease. Am J Epidemiol. 2013;178: 1488–1495. doi:10.1093/aje/kwt156

26. Scott JR, Millar E V., Lipsitch M, Moulton LH, Weatherholtz R, Perilla MJ, et al. Impact of more than a decade of pneumococcal conjugate vaccine use on carriage and invasive potential in native American communities. J Infect Dis. 2012. doi:10.1093/infdis/jir730

27. Weinberger DM, Grant LR, Weatherholtz RC, Warren JL, O’Brien KL, Hammitt LL. Relating Pneumococcal Carriage among Children to Disease Rates among Adults before and after the Introduction of Conjugate Vaccines. Am J Epidemiol. 2016;183: 1055–62. doi:10.1093/aje/kwv283
