# Supplementary material for: Analysing pneumococcal invasiveness using Bayesian models of pathogen progression rates
Source: PLoS Comput Biol. 2022 Feb 17;18(2):e1009389. doi: 10.1371/journal.pcbi.1009389 (PMC8901055; doi:10.1371/journal.pcbi.1009389)
Supplement: S2 Table — These values were generated using the logarithm of the likelihoods calculated within the models. Models are ranked by their expected log pointwise predictive density (ELPD), calculated from the individual pointwise log predictive densities across all observed data points. The ELPD difference column shows the difference between the ELPD of a model and that of the most likely model (this value is zero for the first row, corresponding to the most likely model given the data). The ELPD difference standard error is calculated from the distribution of individual pointwise log predictive densities from the same comparison. (DOCX) [file pcbi.1009389.s037.docx]

| **Model** | **ELPD difference** | **ELPD difference standard error** |
| --- | --- | --- |
| study-adjusted type-specific Poisson | 0.00 | 0.00 |
| study-adjusted type-specific negative binomial | -13.50 | 14.55 |
| type-specific negative binomial | -190.51 | 23.02 |
| study-adjusted negative binomial | -239.41 | 25.27 |
| null negative binomial | -288.29 | 24.84 |
| study-adjusted Poisson | -917.07 | 111.26 |
| type-specific Poisson | -950.42 | 144.80 |
| null Poisson | -1931.64 | 223.91 |
