# Supplementary material for: Analysing pneumococcal invasiveness using Bayesian models of pathogen progression rates
Source: PLoS Comput Biol. 2022 Feb 17;18(2):e1009389. doi: 10.1371/journal.pcbi.1009389 (PMC8901055; doi:10.1371/journal.pcbi.1009389)
Supplement: S3 Table — These values were generated using the logarithm of the likelihoods calculated for the observations of isolates from disease only, as these were more constrained than the modelling of isolate counts from carriage. The table is displayed as described for Table S2. (DOCX) [file pcbi.1009389.s038.docx]

| **Model** | **ELPD difference** | **ELPD difference standard error** |
| --- | --- | --- |
| study-adjusted type-specific negative binomial | 0.00 | 0.00 |
| study-adjusted type-specific Poisson | -15.26 | 11.12 |
| type-specific negative binomial | -162.73 | 12.55 |
| study-adjusted negative binomial | -201.65 | 15.53 |
| null negative binomial | -253.69 | 15.42 |
| type-specific Poisson | -815.05 | 96.09 |
| study-adjusted Poisson | -825.44 | 116.27 |
| null Poisson | -1741.04 | 191.89 |
