# Supplementary material for: Analysing pneumococcal invasiveness using Bayesian models of pathogen progression rates
Source: PLoS Comput Biol. 2022 Feb 17;18(2):e1009389. doi: 10.1371/journal.pcbi.1009389 (PMC8901055; doi:10.1371/journal.pcbi.1009389)
Supplement: S4 Table — Each row corresponds to data simulated from the specified model. All eight models were fitted to each set of simulated data. The table shows the models adjudged to be the first and second best-fitting to each dataset, using bridge sampling. The final column shows the logarithm of the Bayes factor by which the best-fitting model was favoured over the second best-fitting model. (DOCX) [file pcbi.1009389.s039.docx]

| **Simulated model** | **Best-fitting model** | **Second best-fitting model** | **Log(Bayes factor) relative to most likely model** |
| --- | --- | --- | --- |
| null Poisson | null Poisson | null negative binomial | -26.32 |
| null negative binomial | null negative binomial | type-specific negative binomial | -21.24 |
| type-specific Poisson | type-specific Poisson | type-specific negative binomial | -25.15 |
| type-specific negative binomial | type-specific negative binomial | study-adjusted type-specific negative binomial | -25.37 |
| study-adjusted Poisson | study-adjusted Poisson | study-adjusted negative binomial | -41.09 |
| study-adjusted negative binomial | study-adjusted negative binomial | study-adjusted type-specific negative binomial | -26.80 |
| study-adjusted type-specific Poisson | study-adjusted type-specific Poisson | study-adjusted type-specific negative binomial | -33.23 |
| study-adjusted type-specific negative binomial | study-adjusted type-specific negative binomial | study-adjusted negative binomial | -26.87 |
