# Supplementary material for: Analysing pneumococcal invasiveness using Bayesian models of pathogen progression rates
Source: PLoS Comput Biol. 2022 Feb 17;18(2):e1009389. doi: 10.1371/journal.pcbi.1009389 (PMC8901055; doi:10.1371/journal.pcbi.1009389)
Supplement: S5 Table — Models are ranked by their logarithmic marginal likelihoods. The logarithmic Bayes factors were calculated for each model relative to that which was found to be the most likely given the data; hence the value is zero for the first row. (DOCX) [file pcbi.1009389.s040.docx]

| **Model** | **Log(Bayes factor) relative to most likely model** |
| --- | --- |
| study-adjusted type-specific negative binomial | 0.00 |
| study-adjusted type-specific Poisson | -30.92 |
| type-specific negative binomial | -96.18 |
| study-adjusted negative binomial | -119.47 |
| null negative binomial | -142.10 |
| study-adjusted Poisson | -793.66 |
| type-specific Poisson | -840.98 |
| null Poisson | -1838.49 |
