# Supplementary material for: Analysing pneumococcal invasiveness using Bayesian models of pathogen progression rates
Source: PLoS Comput Biol. 2022 Feb 17;18(2):e1009389. doi: 10.1371/journal.pcbi.1009389 (PMC8901055; doi:10.1371/journal.pcbi.1009389)
Supplement: S6 Table — The table is displayed as described for Table S5. (DOCX) [file pcbi.1009389.s041.docx]

| **Model** | **Log(Bayes factor) relative to most likely model** |
| --- | --- |
| study-adjusted type-specific negative binomial | 0.00 |
| study-adjusted type-specific Poisson | -17.03 |
| study-adjusted negative binomial | -17.54 |
| null negative binomial | -28.38 |
| type-specific negative binomial | -36.37 |
| type-specific Poisson | -470.82 |
| study-adjusted Poisson | -945.88 |
| null Poisson | -1814.07 |
