# Supplementary material for: Analysing pneumococcal invasiveness using Bayesian models of pathogen progression rates
Source: PLoS Comput Biol. 2022 Feb 17;18(2):e1009389. doi: 10.1371/journal.pcbi.1009389 (PMC8901055; doi:10.1371/journal.pcbi.1009389)
Supplement: S7 Table — The disease isolates from Portugal came from a mixture of infants and adults, but are tabulated based on them primarily arising from the latter age category. (DOCX) [file pcbi.1009389.s042.docx]

| **Population** | **Vaccine period** | **Carriage study time interval** | **Carriage isolate source** | **Disease isolate source** | **No. of swabs** | **Population of children** | **No. disease isolates from children** | **Population of adults** | **No. disease isolates from adults** | **References** |
| --- | --- | --- | --- | --- | --- | --- | --- | --- | --- | --- |
| Finland | Pre-PCV | 1994 - 1996 | Unvaccinated children <2 years old in Tampere | Unvaccinated children <2 years across Finland | 329 | 120,238 | 143 | - | - | [1,2] |
| Oxford | Pre-PCV | 1994 - 2001 | Unvaccinated healthy  children <5 years old  in Oxford | Unvaccinated  children <5  years old with  IPD in Oxford | 639 | 37,467 | 84 | - | - | [3,4] |
| Portugal | Pre-PCV | January  2001 - December  2003 | Unvaccinated healthy  children <7 years old  in Lisbon and Oeiras | Unvaccinated children (<18 years) and adults (>18 years) across Portugal | 1,170 | 2,071,223 | - | 8,284,894 | 152 | [5,6] |
| Stockholm | Pre-PCV | 1997 - 2004 | Unvaccinated children <7 years old attending day care centres in Stockholm County | Unvaccinated children <18 years old from the Stockholm area | 1,330 | 397,289 | 65 | - | - | [7–10] |
| South Africa | Post-PCV7 | 2009 - 2010 | HIV negative children <13 years old in Soweto and Agincourt | HIV negative children <7 years old in South Africa | 2,674 | 7,187,314 | 515 | - | - | [11–14] |
| USA | Post-PCV7 | 2006 - 2009 | Children <7 years of age in Massachusetts | Children <7 years old in the USA ABCS regions | 1,983 | 1,931,331 | 405 | - | - | [13,15–18] |
| South Africa | Post-PCV13 | 2011 - 2013 | HIV negative children <13 years old in Soweto and Agincourt | HIV negative children <7 years old in South Africa | 2,023 | 7,329,006 | 340 | - | - | [11–14] |

**References**

1. Statistics Finland. Tilastokeskus. 2021.

2. Hanage WP, Kaijalainen TH, Syrjänen RK, Auranen K, Leinonen M, Mäkelä PH, et al. Invasiveness of serotypes and clones of *Streptococcus pneumoniae* among children in Finland. Infect Immun. 2005;73: 431–5. doi:10.1128/IAI.73.1.431-435.2005

3. Office for National Statistics. Mid-1998 Population Estimates: Single year of age and sex for local authorities in England and Wales; estimated resident population. 2004.

4. Brueggemann AB, Griffiths DT, Peto T, Meats E, Crook DW, Spratt BG. Clonal Relationships between Invasive and Carriage *Streptococcus pneumoniae* and Serotype‐ and Clone‐Specific Differences in Invasive Disease Potential. J Infect Dis. 2003;187: 1424–32. doi:10.1086/374624

5. Instituto Nacionalde Estadistica Portugal. Censos 2001. 2002.

6. Sá-Leao R, Pinto F, Aguiar S, Nunes S, Carriço JAJA, Frazao N, et al. Analysis of invasiveness of pneumococcal serotypes and clones circulating in Portugal before widespread use of conjugate vaccines reveals heterogeneous behavior of clones expressing the same serotype. J Clin Microbiol. 2011;49: 1369–75. doi:10.1128/jcm.01763-10

7. Statistics Sweden. Population by region, marital status, age and sex. Year 1968 - 2018. Available: http://www.statistikdatabasen.scb.se/pxweb/en/ssd/START__BE__BE0101__BE0101A/BefolkningNy/?rxid=e550e75b-4cef-4e77-b5e5-83adcc09c97e

8. Normark BH, Christensson B, Sandgren A, Noreen B, Sylvan S, Burman LG, et al. Clonal Analysis of *Streptococcus pneumoniae* Nonsusceptible to Penicillin at Day-Care Centers with Index Cases, in a Region with Low Incidence of Resistance: Emergence of an Invasive Type 35B Clone among Carriers. Microb Drug Resist. 2003;9: 337–344. doi:10.1089/107662903322762761

9. Galanis I, Lindstrand A, Darenberg J, Browall S, Nannapaneni P, Sjöström K, et al. Effects of PCV7 and PCV13 on invasive pneumococcal disease and carriage in Stockholm, Sweden. Eur Respir J. 2016;47: 1208–1218. doi:10.1183/13993003.01451-2015

10. Browall S, Backhaus E, Naucler P, Galanis I, Sjöström K, Karlsson D, et al. Clinical manifestations of invasive pneumococcal disease by vaccine and non-vaccine types. Eur Respir J. 2014;44: 1646–57. doi:10.1183/09031936.00080814

11. Nzenze SA, Von Gottberg A, Shiri T, Van Niekerk N, De Gouveia L, Violari A, et al. Temporal Changes in Pneumococcal Colonization in HIV-infected and HIV-uninfected Mother-Child Pairs Following Transitioning from 7-valent to 13-valent Pneumococcal Conjugate Vaccine, Soweto, South Africa. J Infect Dis. 2015. doi:10.1093/infdis/jiv167

12. Nzenze SA, Shiri T, Nunes MC, Klugman KP, Kahn K, Twine R, et al. Temporal changes in pneumococcal colonization in a rural African community with high HIV prevalence following routine infant pneumococcal immunization. Pediatr Infect Dis J. 2013;32: 1270–1278. doi:10.1097/01.inf.0000435805.25366.64

13. Gladstone RA, Lo SW, Lees JA, Croucher NJ, van Tonder AJ, Corander J, et al. International genomic definition of pneumococcal lineages, to contextualise disease, antibiotic resistance and vaccine impact. EBioMedicine. 2019;43: 338–346. doi:10.1016/j.ebiom.2019.04.021

14. Statistics South Africa. Mid-year population estimates: 2009-2013. 2013.

15. Huang SS, Hinrichsen VL, Stevenson AE, Rifas-Shiman SL, Kleinman K, Pelton SI, et al. Continued Impact of Pneumococcal Conjugate Vaccine on Carriage in Young Children. Pediatrics. 2009;124: e1-11. doi:10.1542/peds.2008-3099

16. Croucher NJ, Finkelstein JA, Pelton SI, Mitchell PK, Lee GM, Parkhill J, et al. Population genomics of post-vaccine changes in pneumococcal epidemiology. Nat Genet. 2013;45: 656–663. doi:10.1038/ng.2625

17. Wroe PC, Lee GM, Finkelstein JA, Pelton SI, Hanage WP, Lipsitch M, et al. Pneumococcal carriage and antibiotic resistance in young children before 13-valent conjugate vaccine. Pediatr Infect Dis J. 2012;31: 249–254. doi:10.1097/INF.0b013e31824214ac

18. Centres for Disease Prevention and Control. Active Bacterial Core surveillance matrix. 2018. Available: https://www.cdc.gov/abcs/downloads/abcs-surveillance-matrix.pdf
