# Supplementary material for: Analysing pneumococcal invasiveness using Bayesian models of pathogen progression rates
Source: PLoS Comput Biol. 2022 Feb 17;18(2):e1009389. doi: 10.1371/journal.pcbi.1009389 (PMC8901055; doi:10.1371/journal.pcbi.1009389)
Supplement: S8 Table — These values were generated using the logarithm of the likelihoods calculated for the observations of isolates from carriage and disease. The table is displayed as described for Table S2. (DOCX) [file pcbi.1009389.s043.docx]

| **Model** | **ELPD difference** | **ELPD difference standard error** |
| --- | --- | --- |
| type-specific strain-modified Poisson | 0.00 | 0.00 |
| strain- and serotype-specific Poisson | -1.56 | 3.57 |
| strain-specific type-modified Poisson | -4.77 | 3.50 |
| type-specific strain-modified negative binomial | -26.97 | 3.34 |
| strain-specific type-modified negative binomial | -37.88 | 4.89 |
| type-specific negative binomial | -41.08 | 5.91 |
| strain-specific Poisson | -43.61 | 18.76 |
| type-specific Poisson | -45.21 | 14.48 |
| strain- and type-specific negative binomial | -48.23 | 6.02 |
| strain-specific negative binomial | -61.00 | 6.81 |
