# Supplementary material for: Analysing pneumococcal invasiveness using Bayesian models of pathogen progression rates
Source: PLoS Comput Biol. 2022 Feb 17;18(2):e1009389. doi: 10.1371/journal.pcbi.1009389 (PMC8901055; doi:10.1371/journal.pcbi.1009389)
Supplement: S9 Table — These values were generated using the logarithm of the likelihoods calculated for the observations of isolates from disease only. The table is displayed as described for Table S2. (DOCX) [file pcbi.1009389.s044.docx]

| **Model** | **ELPD difference** | **ELPD difference standard error** |
| --- | --- | --- |
| type-specific strain-modified Poisson | 0.00 | 0.00 |
| strain- and type-specific Poisson | -0.57 | 2.66 |
| strain-specific type-modified Poisson | -3.29 | 2.34 |
| type-specific strain-modified negative binomial | -21.14 | 2.48 |
| type-specific negative binomial | -32.08 | 5.14 |
| strain-specific type-modified negative binomial | -32.94 | 4.17 |
| strain-specific Poisson | -36.85 | 15.83 |
| strain- and type-specific negative binomial | -41.17 | 5.35 |
| type-specific Poisson | -44.04 | 13.05 |
| strain-specific negative binomial | -51.28 | 5.63 |
