# Supplementary material for: Analysing pneumococcal invasiveness using Bayesian models of pathogen progression rates
Source: PLoS Comput Biol. 2022 Feb 17;18(2):e1009389. doi: 10.1371/journal.pcbi.1009389 (PMC8901055; doi:10.1371/journal.pcbi.1009389)
Supplement: S10 Table — The table is displayed as described for Table S5. (DOCX) [file pcbi.1009389.s045.docx]

| **Model** | **Log(Bayes factor) relative to best-fitting model** |
| --- | --- |
| type-specific strain-modified Poisson | 0.00 |
| type-specific negative binomial | -5.59 |
| type-specific strain-modified negative binomial | -5.99 |
| type-specific Poisson | -24.61 |
| strain-specific type-modified Poisson | -25.27 |
| strain-specific serotype-modified negative binomial | -29.98 |
| strain- and type-specific Poisson | -33.29 |
| strain-specific negative binomial | -33.94 |
| strain- and type-specific negative binomial | -37.33 |
| strain-specific Poisson | -49.46 |
