# Supplementary material for: Analysing pneumococcal invasiveness using Bayesian models of pathogen progression rates
Source: PLoS Comput Biol. 2022 Feb 17;18(2):e1009389. doi: 10.1371/journal.pcbi.1009389 (PMC8901055; doi:10.1371/journal.pcbi.1009389)
Supplement: S11 Table — In this analysis, the carriage sample size for three studies (Finland pre-PCV, Oxford pre-PCV and Stockholm pre-PCV) was increased 100-fold, to test the sensitivity of the model comparisons to the uncertainty in this parameter (Text S2). The table is displayed as described for Table S5. (DOCX) [file pcbi.1009389.s046.docx]

| **Model** | **Log(Bayes factor) relative to best-fitting model** |
| --- | --- |
| type-specific strain-modified Poisson | 0.00 |
| type-specific negative binomial | -5.63 |
| type-specific strain-modified negative binomial | -5.94 |
| type-specific Poisson | -24.70 |
| strain-specific type-modified Poisson | -25.28 |
| strain-specific serotype-modified negative binomial | -29.67 |
| strain- and type-specific Poisson | -32.86 |
| strain-specific negative binomial | -33.49 |
| strain- and type-specific negative binomial | -36.48 |
| strain-specific Poisson | -49.40 |
