# Supplementary material for: Analysing pneumococcal invasiveness using Bayesian models of pathogen progression rates
Source: PLoS Comput Biol. 2022 Feb 17;18(2):e1009389. doi: 10.1371/journal.pcbi.1009389 (PMC8901055; doi:10.1371/journal.pcbi.1009389)
Supplement: S12 Table — The exclusion of strain and serotype combinations represented by fewer than ten isolates, summed across carriage and disease, meant that some within-serotype differences between strains were not tested in this analysis. (DOCX) [file pcbi.1009389.s047.docx]

| **Previous publication** | **Serotype** | **Low invasiveness genotype** | **Low invasiveness strain** | **High invasiveness genotype** | **High invasiveness strain** | **Results from this study** |
| --- | --- | --- | --- | --- | --- | --- |
| Sá-Leão *et al* 2011 [1] | 3 | ST180 | GPSC12 | ST156 | GPSC6 | GPSC6 was estimated to have higher invasiveness, albeit with overlapping credibility intervals |
| Gladstone *et al* 2019 [2] | 6A | CC172 | GPSC5 | CC1094 | GPSC41 | GPSC41 invasiveness was found to be significantly higher than GPSC5 |
| Sá-Leão *et al* 2011 [1] | 6A | ST460, ST315, ST1879 | GPSC47, GPSC64, ST1879 | - | - | Other GPSCs of this serotype were not tested in adults |
| Sá-Leão *et al* 2011 [1] | 6B | ST176, ST315 | GPSC24, GPSC47 | - | - | Other GPSCs of this serotype were not tested in adults |
| Hanage *et al* 2005 [3] | 6B | - | - | ST138 | GPSC24 | GPSC24 was found to be more invasive than other 6B GPSCs |
| Hanage *et al* 2005 [3] | 7F | - | - | ST191 | GPSC15 | Not tested |
| Sá-Leão *et al* 2011 [1] | 11A | ST62 | GPSC3 | - | - | Not tested in adults |
| Hanage *et al* 2005 [3] | 11A | ST62 | GPSC3 | - | - | Estimated to have similar invasiveness as GPSC22 |
| Gladstone *et al* 2019 [2] | 14 | CC63 | GPSC9 | CC15 | GPSC18 | GPSC18 invasiveness was estimated to be higher than that of GPSC9, albeit with slightly overlapping credibility intervals |
| Sá-Leão *et al* 2011 [1] | 14 | - | - | ST156 | GPSC6 | GPSC6 was found to be more invasive than GPSC9, albeit with overlapping credibility intervals, in adults |
| Hanage *et al* 2005 [3] | 14 | - | - | ST156 | GPSC6 | GPSC6 was found to be more invasive than GPSC9, albeit with overlapping credibility intervals |
| Brueggemann *et al* 2003 [4] | 14 | - | - | ST9 | GPSC18 | GPSC18 was found to be more invasive than GPSC9, albeit with overlapping credibility intervals |
| Brueggemann *et al* 2003 [4] | 14 | - | - | ST124 | GPSC39 | GPSC39 was estimated to be more invasive than GPSC9, albeit with overlapping credibility intervals |
| Hanage *et al* 2005 [3] | 14 | - | - | ST124 | GPSC39 | GPSC39 was estimated to be more invasive than GPSC9, albeit with overlapping credibility intervals |
| Gladstone *et al* 2019 [2] | 16F | CC4088 | GPSC33 | CC30 | GPSC46 | Not tested |
| Brueggemann *et al* 2003 [4] | 18C | - | - | ST113 | GPSC50 | Estimated invasiveness is similar to that of GPSC67 and GPSC68 |
| Gladstone *et al* 2019 [2] | 19F | CC347 | GPSC21 | CC320 | GPSC1 | GPSC1 invasiveness was estimated to be significantly higher than that of GPSC21 |
| Sá-Leão *et al* 2011 [1] | 19F | ST177 | GPSC44 | - | - | Not tested in adults |
| Hanage *et al* 2005 [3] | 19F | ST485 | GPSC175 | - | - | Low invasiveness, with overlapping credibility intervals with other GPSCs |
| Sá-Leão *et al* 2011 [1] | 19A | ST81 | GPSC16 | ST193, ST230 | GPSC11, GPSC10 | Not tested in adults |
| Hanage *et al* 2005 [3] | 19A | - | - | ST482 | ST482 | Higher invasiveness than GPSC17, albeit with overlapping credibility intervals |
| Sá-Leão *et al* 2011 [1] | 22F | - | - | ST443 | ST443 | Not tested in adults |
| Gladstone *et al* 2019 [2] | 23B | CC439 | GPSC7 | CC172 | GPSC5 | Not tested, although GPSC5 was found to be more invasive that GPSC7 in serotype 23A |
| Sá-Leão *et al* 2011 [1] | 23F | ST176, ST439 | GPSC24, GPSC7 | - | - | GPSC7 was estimated to have lower invasiveness than GPSC5 in adults, albeit with overlapping credibility intervals |
| Sá-Leão *et al* 2011 [1] | 34 | ST1439 | GPSC45 | - | - | Not tested in adults |

**References**

1. Sá-Leao R, Pinto F, Aguiar S, Nunes S, Carriço JAJA, Frazao N, et al. Analysis of invasiveness of pneumococcal serotypes and clones circulating in Portugal before widespread use of conjugate vaccines reveals heterogeneous behavior of clones expressing the same serotype. J Clin Microbiol. 2011;49: 1369–75. doi:10.1128/jcm.01763-10

2. Gladstone RA, Lo SW, Lees JA, Croucher NJ, van Tonder AJ, Corander J, et al. International genomic definition of pneumococcal lineages, to contextualise disease, antibiotic resistance and vaccine impact. EBioMedicine. 2019;43: 338–346. doi:10.1016/j.ebiom.2019.04.021

3. Hanage WP, Kaijalainen TH, Syrjänen RK, Auranen K, Leinonen M, Mäkelä PH, et al. Invasiveness of serotypes and clones of Streptococcus pneumoniae among children in Finland. Infect Immun. 2005;73: 431–5. doi:10.1128/IAI.73.1.431-435.2005

4. Brueggemann AB, Griffiths DT, Peto T, Meats E, Crook DW, Spratt BG. Clonal Relationships between Invasive and Carriage *Streptococcus pneumoniae* and Serotype‐ and Clone‐Specific Differences in Invasive Disease Potential. J Infect Dis. 2003;187: 1424–32. doi:10.1086/374624
